# The BBX gene family in Moso bamboo (*Phyllostachys edulis*): identification, characterization and expression profiles

**DOI:** 10.1186/s12864-021-07821-w

**Published:** 2021-07-13

**Authors:** Ruifang Ma, Jialu Chen, Bin Huang, Zhinuo Huang, Zhijun Zhang

**Affiliations:** 1 State Key Laboratory of Subtropical Forest Cultivation, Zhejiang A&F University, Lin’an, Zhejiang 311300 Hangzhou, China; 2 School of Forestry and Biotechnology, Zhejiang A&F University, Lin’an, Zhejiang 311300 Hangzhou, China

**Keywords:** Moso bamboo, Gene family, Expression analysis, Characterization

## Abstract

**Background:**

The BBX (B-box) family are zinc finger protein (ZFP) transcription factors that play an essential role in plant growth, development and response to abiotic stresses. Although BBX genes have been characterized in many model organisms, genome-wide identification of the BBX family genes have not yet been reported in Moso bamboo (*Phyllostachys edulis*), and the biological functions of this family remain unknown.

**Result:**

In the present study, we identified 27 *BBX* genes in the genome of Moso bamboo, and analysis of their conserved motifs and multiple sequence alignments revealed that they all shared highly similar structures. Additionally, phylogenetic and homology analyses indicated that *PeBBX* genes were divided into three clusters, with whole-genome duplication (WGD) events having facilitated the expansion of this gene family. Light-responsive and stress-related *cis*-elements were identified by analyzing *cis*-elements in the promoters of all *PeBBX* genes. Short time-series expression miner (STEM) analysis revealed that the *PeBBX* genes had spatiotemporal-specific expression patterns and were likely involved in the growth and development of bamboo shoots. We further explored the downstream target genes of *PeBBXs*, and GO/KEGG enrichment analysis predicted multiple functions of BBX target genes, including those encoding enzymes involved in plant photosynthesis, pyruvate metabolism and glycolysis/gluconeogenesis.

**Conclusions:**

In conclusion, we analyzed the *PeBBX* genes at multiple different levels, which will contribute to further studies of the BBX family and provide valuable information for the functional validation of this family.

**Supplementary Information:**

The online version contains supplementary material available at 10.1186/s12864-021-07821-w.

## Background

Transcription factors (TFs) are essential regulatory proteins that can activate or repress the expression of their target genes and thus play an important regulatory role in all eukaryotes [1]. This regulation is achieved through specific interactions between the DNA-binding domain of the TF and the promoter of the target gene. These interactions are affected by TF transcriptional activation domains, oligomerization sites, nuclear localization signals and DNA binding domains [2–4]. Zinc finger TF members are divided into several subfamilies, based on their structural and functional characteristics. Among them, B-box (BBX) zinc finger proteins have received considerable attention in recent years due to their diverse functions [5].

BBX are a class of zinc finger protein TFs with one or two BBX structural domains, with some members also possessing CCT (conserved carboxy-terminal) structural domains. The most distinctive feature of BBX TFs is the presence of one BBX at the N-terminus or two BBX modules in tandem, sometimes with a CCT (CONSTANS, CCMike and TOC) domain, at the C-terminus [6]. The BBX structure consists of 1–2 BBX modules with a length of 40 amino acid residues. Based on the similar sequences of the two modules, and the spacing characteristics with zinc-binding residues, BBXs can be classified into two types: BBX l (B1) and BBX 2 (B2). The zinc finger type of B1 is C-X2-C-X7-8-C-X2-D-X-A-X-L-C-X2-C- D-X3-HB, while the zinc finger type of B2 is C-X2-C-X3-P-X4-C-X2-D-X3-L-C-X2-C-D-X3-H [7, 8].

The BBX proteins have unique tertiary structures that are stabilized by binding Zn ions [9]. Studies have shown that the BBX domain regulates protein-protein interactions as well as transcriptional processes [10–12]. BBX proteins have been shown to affect seedling development by integrating plant phytochromes and light signals sensed by cryptic photoreceptors through the COP1 and HY5 signaling pathways. In addition, some BBX proteins are involved in the photoperiodic pathway of flowering [13, 14]. Sequence alignment analysis of BBX proteins suggests that the CCT structural domain of some BBX proteins are also highly conserved and functional [6], typically consisting of 42–43 amino acids [15]. Both *Arabidopsis* and rice each possess 17 members that have a CCT domain at their C-terminus, which plays a vital role in transcriptional regulation and nuclear protein transport [16, 17]. The *Arabidopsis* regulator of photoperiodic flowering CO (CONSTAVS), or BBX1, regulates FT (FLOWERING LOCUS T) expression to control the flowering pathway by acting directly on the promoter of FT [15]. In addition, the CCT structural domain includes nuclear localization signals, which play an active role in the cytosolic localization of BBX TFs [18, 16, 19].

Plants adapt to various environmental changes by altering their gene regulatory networks [20]. BBX TFs are induced in response to different abiotic stresses and are important candidates for enhancing abiotic stress tolerance in plants. In *Arabidopsis*, *COLI* (*BBX2*) is consistently up-regulated during long periods of cold acclimation [21] and *BBX32* in *Arabidopsis* is responsive to methyl jasmonate (MeJA) hormone stress. In chrysanthemum, *CmBBX24* improves tolerance to abiotic stresses, especially drought stress and low-temperature stress [22]. In banana, transcripts of the *MaCOL1* gene were shown to accumulate rapidly under cold treatment [23].

Bamboo is one of the most important non-timber forest products globally [24]. Moso bamboo (*Phyllostachys edulis, P. edulis*) is fast-growing, easy to establish, and has both ecological and social benefits [25]. Due to these beneficial features, bamboo is widely used for wood, paper, artwork and food [26, 27]. In China, several species of bamboo are grown for dual use, with shoots being harvested for consumption while mature wood is used for timber [28].

The development and distribution of Moso bamboo are limited by unfavorable climatic and environmental conditions [29]. Genomic studies have the potential to result in dramatic increases in plant stress tolerance [30], but the previously published genome of Moso bamboo has not been sufficiently explored for this purpose. The new assembly of the Moso bamboo genome offers an opportunity to improve our understanding of the abundance, distribution and expansion of bamboo *BBX* genes [31, 32]. In this study, we identified 27 genes encoding of Moso bamboo BBX family (*PeBBX*) and investigated their structural domains, amplification patterns, and evolutionary relationships.

## Results

### Identification and characterization of *PeBBX* genes in *P. edulis*

Putative *BBX* candidate genes were obtained from an HMMER3 search of the bamboo protein database using the plant BBX-type model (Pfam PF00643) with an E-value threshold of ≤ 10^− 5^. We removed redundant genes and verified the presence of conserved domains and motifs to arrive at a final set of 27 *BBX* family members. The genes were renamed *PeBBX01*–*PeBBX27* based on their positions on chromosomal scaffolds (Table 1). Proteins encoded by the 27 *PeBBX* genes contained 70 (*PeBBX09*) to 446 (*PeBBX17*) amino acids, and their MWs ranged from 7165.31 (*PeBBX09*) to 48231.99 (*PeBBX17*). Their predicted pIs ranged from 4.91 (*PeBBX04*/*PeBBX13*) to 8.49 (*PeBBX15*). Aliphatic amino acid indices showed that the thermal stability of the proteins ranged from 55.59 to 84.00, indicating that differences in their thermal stability were relatively minor. The grand average of hydropathicity (GRAVY) scores of all BBX proteins were negative, suggesting that they were hydrophilic proteins. We predicted the subcellular localization of the proteins encoded by the *PeBBX* genes and found that all were likely to localize to the nucleus.

**Table 1:** Physicochemical properties of *PeBBXs* genes

ID	ID Rename	Deduced amino acids	Chromosomal locus	Molecular weight	Theoretical pI	Aliphatic index	Grand average of hydropathicity (GRAVY)	Signal Peptide	Subcellular Localization Prediction
PH02Gene35355.t1	*PeBBX01*	211	scaffold3	23327.18	5.9	62.84	-0.632	No	Nucleus
PH02Gene27822.t1	*PeBBX02*	327	scaffold3	34238.22	5.22	71.01	-0.176	No	Nucleus
PH02Gene39681.t1	*PeBBX03*	259	scaffold3	27821.24	5.7	65.33	-0.235	No	Nucleus
PH02Gene16459.t1	*PeBBX04*	274	scaffold3	29368.37	4.91	69.23	-0.319	Yes	Nucleus
PH02Gene45949.t1	*PeBBX05*	439	scaffold3	47906.54	5.96	66.31	-0.566	No	Nucleus
PH02Gene19468.t1	*PeBBX06*	369	scaffold6	39699.18	4.95	70.11	-0.390	No	Nucleus
PH02Gene05295.t1	*PeBBX07*	418	scaffold6	45562.27	5.87	65.45	-0.561	No	Nucleus
PH02Gene19765.t2	*PeBBX08*	284	scaffold6	29897.52	5.33	75.46	-0.250	No	Nucleus
PH02Gene25549.t1	*PeBBX09*	70	scaffold8	7165.31	5.47	84.00	0.137	No	Nucleus
PH02Gene21910.t1	*PeBBX10*	382	scaffold8	41886.71	5.05	67.49	-0.410	No	Nucleus
PH02Gene02845.t1	*PeBBX11*	272	scaffold8	28909.61	5.27	76.58	-0.196	No	Nucleus
PH02Gene42827.t1	*PeBBX12*	321	scaffold9	34325.22	5.29	56.07	-0.429	No	Nucleus
PH02Gene23282.t1	*PeBBX13*	212	scaffold12	21867.18	4.91	70.47	-0.228	No	Nucleus
PH02Gene32457.t1	*PeBBX14*	346	scaffold14	36826.24	5.64	62.17	-0.294	No	Nucleus
PH02Gene09921.t1	*PeBBX15*	393	scaffold15	43019.72	8.49	65.67	-0.557	No	Nucleus
PH02Gene20422.t1	*PeBBX16*	344	scaffold16	36537.83	5.33	61.72	-0.328	No	Nucleus
PH02Gene08112.t1	*PeBBX17*	446	scaffold17	48231.99	7.05	67.26	-0.551	No	Nucleus
PH02Gene29322.t1	*PeBBX18*	261	scaffold17	27900.11	5.26	62.99	-0.306	No	Nucleus
PH02Gene28332.t1	*PeBBX19*	326	scaffold17	34492.53	5.25	70.37	-0.271	No	Nucleus
PH02Gene14035.t1	*PeBBX20*	257	scaffold17	27748.19	5.06	75.68	-0.295	No	Nucleus
PH02Gene08231.t1	*PeBBX21*	322	scaffold18	34437.41	5.65	55.59	-0.457	No	Nucleus
PH02Gene13905.t1	*PeBBX22*	394	scaffold21	43179.69	5.94	66.50	-0.589	No	Nucleus
PH02Gene35510.t1	*PeBBX23*	249	scaffold23	26905.87	5.28	64.38	-0.427	No	Nucleus
PH02Gene06669.t1	*PeBBX24*	332	scaffold23	34866.85	5.25	65.57	-0.308	No	Nucleus
PH02Gene11378.t1	*PeBBX25*	256	scaffold23	27484.9	5.01	74.77	-0.286	No	Nucleus
PH02Gene03202.t1	*PeBBX26*	255	scaffold24	27165.64	4.98	77.02	-0.177	No	Nucleus
PH02Gene02031.t1	*PeBBX27*	252	scaffold24	27505.72	5.04	67.86	-0.378	No	Nucleus

### Phylogenetic analysis

**Fig. 1:**
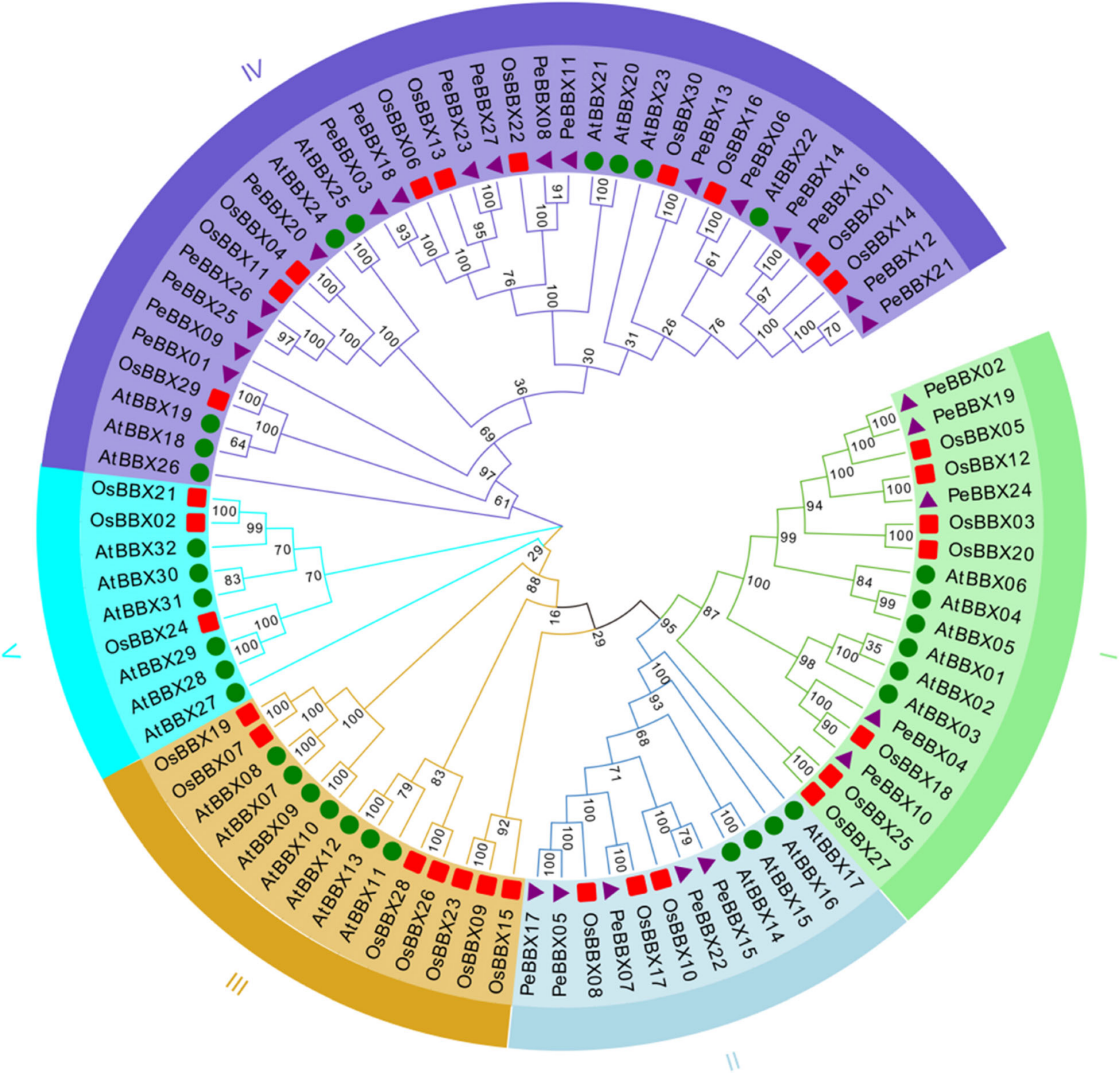
Phylogenetic tree analysis of BBX sequences. The full-length amino acid sequences of 89 BBX proteins were used to construct the phylogenetic tree using MEGA7.0 with the neighbor-joining (NJ) method. The number at the branch represents the confidence value obtained by 100 bootstrap tests. AtBBX represents BBX protein sequence of *Arabidopsis thaliana* and OsBBX represents BBX protein sequence of rice

Previous studies have shown that *OsBBX* genes can be divided into five subfamilies [9]. A NJ phylogenetic tree was constructed based on BBX protein sequences from bamboo, rice and *A. thaliana* to demonstrate the phylogenetic relationships between Moso bamboo and Gramineae plants as well as dicotyledonous plants (Additional file 4: Table S1). The sequences of *A. thaliana* were found to be distributed in subclade I, subclade II and subclade IV. However, in subclade III and subclade V, only sequences of *A. thaliana* and rice were found. In subclade IV, Moso bamboo had the highest number of PeBBX sequences, while subclade I and subclade II contained five PeBBX sequences. Moreover, the BBX of Moso bamboo and the BBX of rice clustered in one group (Fig. 1) and were more closely related.

### Gene structure, conserved domains, motifs and sequence analysis

**Fig. 2:**
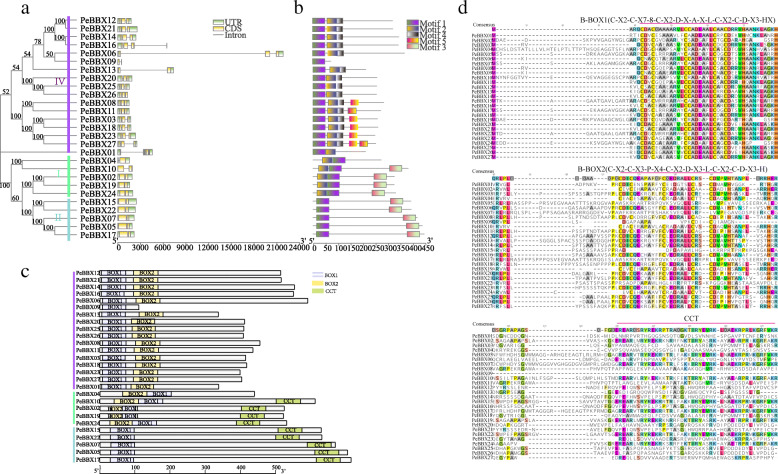
Gene structure, motifs, conserved structural domains, compositions and multiple sequence alignment analysis of the *PeBBX* gene family. **a**. Intron-exon structure of the *PeBBX* genes. Yellow boxes represent exons (CDS), black lines represent introns and green boxes represent the 5’ and 3’ untranslated regions. **b**. Distribution of conserved motifs in PeBBX proteins. The scale bar at the bottom indicates the protein lengths, and sequence logos for each conserved motif are shown on the right. **c**. Conserved domain predictions for the 27 PeBBX proteins. The length of each protein sequence is represented by the grey bars, and colored boxes represent conserved domains. Green boxes represent CCT domains, light purple boxes indicate BOX1 domains and yellow boxes indicate BOX2 domains. d Multiple sequence alignment of PeBBX protein sequences. The number above the sequence indicates the location of the amino acids in the BBX proteins. The key domains are highlighted, and the domain names are shown at the top of the sequence. Protein sequence names are shown on the left

To gain insight into the structural features of *PeBBX* genes in Moso bamboo, we compared their genomic DNA sequences to determine the number of introns and exons within each gene. The gene structures of all 27 *PeBBX* genes were shown in Fig. 2a. Subclade I and subclade II were found to have similar gene structures, with a short overall length and only one intron. After comparing the motifs of subclades I, II and IV, we found that motif 1 of subfamily IV and subfamily II was in the more N-terminal position, while motif 2 of subfamily I was in the more N-terminal position (Fig. 2b). After combining these results with the analysis of conserved structural domains (Fig. 2c), it is clear that motif 3 formed the CCT structural domain, and motif 1 formed the BOX1 structural domain. In contrast, motif 2 formed BOX2 structural domain, and the BOX structural domain was composed of motif 1 and motif 2 together.

To analyze the sequence characteristics of PeBBX proteins, we used the CDD website to predict the structural domains in the full-length sequence of PeBBX proteins, revealing that the subclade IV had 17 members, nearly all of which had two BOX structural domains (BOX1 and BOX2). PeBBX09 was the only protein sequence in subclade IV that had only one BOX1 structural domain. In addition, the PeBBX sequences of the subclade II all possessed one BOX structural domain and one CCT structural domain. In contrast, the PeBBX sequences of the subclade IV all contained two BOX structural domains and one CCT structural domain, with the exception of PeBBX04.

To investigate the prevalence and locations of conserved protein domains, we created a multiple sequence alignment of 27 PeBBX proteins. At the N-terminus, all BBX family members contained a highly conserved BOX domain that consisted of approximately 30 amino acids (Fig. 2d). Some of the BBX sequences also had a CCT domain at the C-terminus.

### Chromosomal location and gene duplication of *PeBBX* genes

**Fig. 3:**
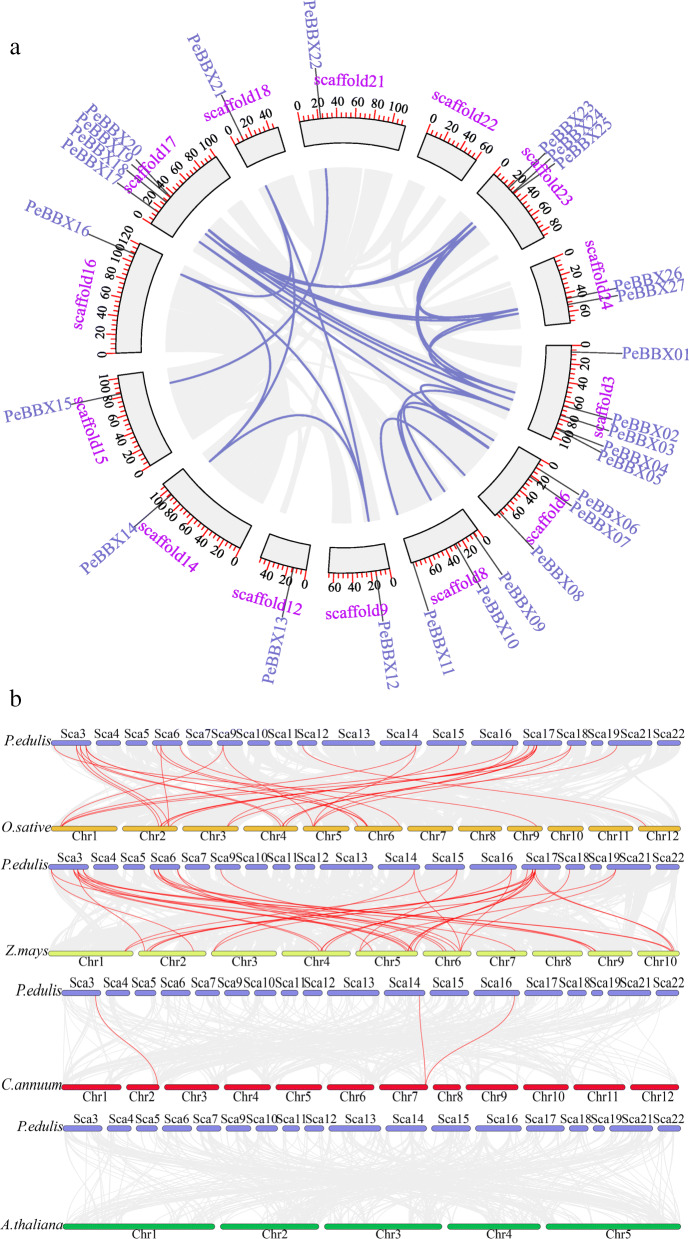
Chromosomal location and duplicated genes among *PeBBX* genes. **a** Intraspecific colinearity analysis. A total of 27 *PeBBXs* were mapped onto the chromosomes on the basis of their physical location. Chromosome numbers (scaffold1- scaffold24) are distributed in the outer circle, the blue lines indicate duplicated *PeBBX* gene pairs. **b** Analysis of collinearity between different species. The gray lines indicate duplicated blocks, while the red lines indicate duplicated BBX gene pairs. Chromosome numbers are at the bottom of each chromosome

The *PeBBX* genes were unequally distributed across the 11 chromosome scaffolds of Moso bamboo (Additional file 1: Figure S1). The largest number was found on scaffold 3 (5), followed by scaffold 17 (4), scaffolds 6 (3), 8 (3), 23 (3) and 2 *PeBBX* genes were mapped on scaffold 24. All other chromosomes contained a single *PeBBX* gene. In all species, gene duplication events are prevalent, and they can produce new functional genes and drive the evolution of species. Therefore, we used MCScanX genomic co-frequency analysis to explore duplications within the *BBX* gene family (Fig. 3a), revealing 30 segmental duplications in in the whole genome of Moso bamboo.

Monocotyledonous plants, including rice and maize, and herbaceous plants, including pepper and *Arabidopsis*, were selected for interspecific collinearity analysis by BLAST comparison of BBX homologous sequences. This analysis revealed that the *BBX* genes in Moso bamboo and rice were highly homologous, indicating that they most likely had similar functions. In the model plant *A. thaliana*, no homology was identified with the *BBX* genes of Moso bamboo. In addition, many highly homologous genes of *BBX* in Moso bamboo were identified in maize, and three homologous genes were identified in pepper (Fig. 3b).

Based on the comparative sequence similarity of BBX family, among which 24 Moso bamboo homologous gene pairs were found, Ka/Ks ratios of the 24 Moso bamboo *BBX* gene pairs were calculated, and the Ks values were used to calculate the time of duplication events of Moso bamboo *BBX* genes. We performed Ka/Ks (evolutionary selection pressure) analysis on 24 *PeBBX* homologous gene pairs in Moso bamboo, and found that among these 24 pairs of homologous genes, *PeBBX04*/*PeBBX10* had the lowest Ka/Ks ratio (0.1734), followed by *PeBBX19*/*PeBBX02* (0.2422), while *PeBBX20*/*PeBBX25* had the highest ratio (0.4798). The Ka/Ks ratio of all Moso bamboo *BBX* homologs were less than 1, indicating a strong purifying selection in the evolution of the Moso bamboo *BBX* gene family (Table 2). The Ks values obtained during this analysis then used to calculate the duplication time of Moso bamboo *BBX* genes during evolution. This analysis results showed that the replication time of Moso bamboo *BBX* genes primarily occurred between 9 and 75 million years ago (mya), with nine gene pair replication events occurring in roughly 9–17 mya.

**Table 2:** Ka/Ks values of homologous *PeBBX* gene pairs

Duplicate gene pair	Ka	Ks	Ka/Ks	Purify Selection	Duplication type	Time (Mya^a^)
*PeBBX14*/*PeBBX16*	0.042104596	0.152056047	0.276901819	Yes	segmental	11.69661899
*PeBBX14*/*PeBBX21*	0.19628168	0.448756769	0.437389905	Yes	segmental	34.51975143
*PeBBX14*/*PeBBX12*	0.182756627	0.446713424	0.4091138	Yes	segmental	34.36257111
*PeBBX15*/*PeBBX22*	0.052573616	0.202958669	0.259036072	Yes	segmental	15.6122053
*PeBBX16*/*PeBBX21*	0.176648673	0.441908756	0.399740151	Yes	segmental	33.9929812
*PeBBX16*/*PeBBX12*	0.173011971	0.46856959	0.369234315	Yes	segmental	36.04381459
*PeBBX17*/*PeBBX07*	0.216882382	0.658188855	0.329513908	Yes	segmental	50.62991194
*PeBBX18*/*PeBBX23*	0.170545595	0.379913416	0.448906482	Yes	segmental	29.22410896
*PeBBX19*/*PeBBX24*	0.165046414	0.522000187	0.316180756	Yes	segmental	40.15386055
*PeBBX20*/*PeBBX25*	0.13529533	0.281953784	0.479849316	Yes	segmental	21.68875262
*PeBBX17*/*PeBBX05*	0.054743612	0.179281304	0.305350367	Yes	segmental	13.79086958
*PeBBX18*/*PeBBX03*	0.041517811	0.139739347	0.297108955	Yes	segmental	10.74918051
*PeBBX19*/*PeBBX02*	0.043371875	0.179063587	0.242214932	Yes	segmental	13.77412206
*PeBBX18*/*PeBBX27*	0.139056615	0.335375829	0.414629209	Yes	segmental	25.79814069
*PeBBX20*/*PeBBX26*	0.136471916	0.295718856	0.461492101	Yes	segmental	22.74760428
*PeBBX21*/*PeBBX12*	0.040608208	0.131673581	0.308400577	Yes	segmental	10.12873698
*PeBBX27*/*PeBBX03*	0.141112234	0.352596186	0.40020919	Yes	segmental	27.12278352
*PeBBX24*/*PeBBX02*	0.167787595	0.475482406	0.35287866	Yes	segmental	36.57556972
*PeBBX23*/*PeBBX03*	0.165269986	0.429437165	0.384852546	Yes	segmental	33.03362806
*PeBBX23*/*PeBBX27*	0.051435606	0.126234197	0.40746174	Yes	segmental	9.710322865
*PeBBX06*/*PeBBX09*	0.067830454	0.194146225	0.349378174	Yes	segmental	14.93432503
*PeBBX08*/*PeBBX11*	0.056814836	0.148800797	0.381818089	Yes	segmental	11.44621513
*PeBBX05*/*PeBBX07*	0.205355044	0.547240162	0.375255799	Yes	segmental	42.09539711
*PeBBX04*/*PeBBX10*	0.16485542	0.950757381	0.173393784	Yes	segmental	73.13518318

^a^million years ago

### *Cis*-element analysis of *PeBBXs*

**Fig. 4:**
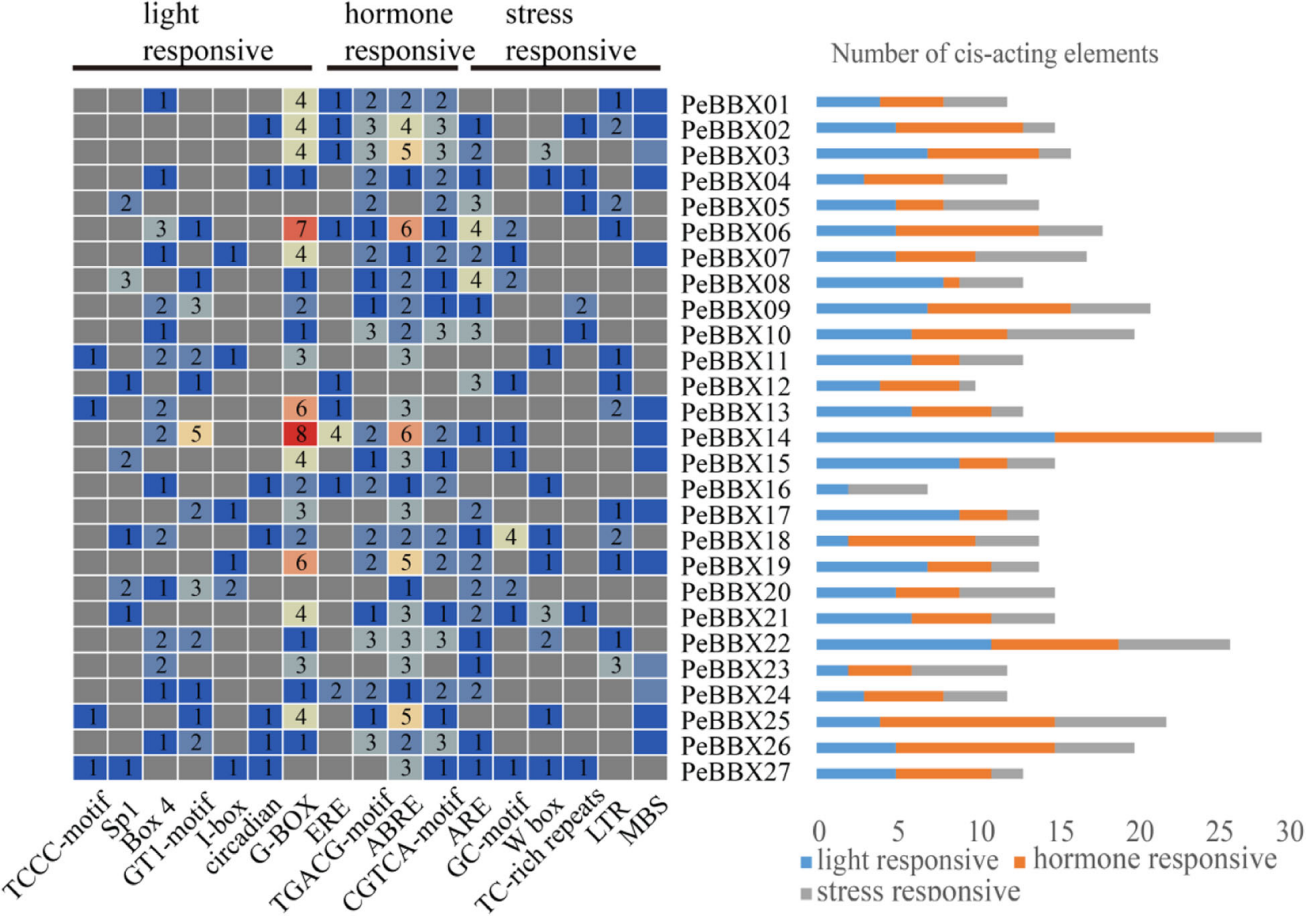
*Cis*-element analysis of *PeBBXs. *Information about the promoters of PeBBX genes. The graph on the left shows cis-acting element enrichment, with different colors indicating different element numbers. The chart on the right is a proportional map, the blue rectangle represents light responsive cis-elements, the orange rectangle represents hormone responsive cis-elements, and the gray rectangle represents stress responsive cis-elements

Sequence analysis of the promoter regions of *PeBBX* genes via the PlantCARE website showed 431 associated *cis*-acting elements (Fig. 4). These included 152 *cis*-elements related to light response regulation. In addition, there were hormone-responsive elements, including Ethylene response element (ERE), abscisic acid (ABA) responsive elements (ABRE, ABA responsiveness) and MeJA acid responsive elements (CGTCA-motif and TGACG-motif). There were also other stress responsive elements, anaerobic response regulatory elements (ARE, anaerobic responsiveness), drought-related regulatory elements (MBS), low-temperature response elements (LTR, low-temperature responsiveness), defense and stress responsiveness (TC-rich motif) and wounding and pathogen responsiveness (W-box). It was noteworthy that each gene had a unique composition of cis-acting elements (Additional file 2: Figure S2, Additional file 5: Table S2), with *PeBBX06*, *PeBBX13* and *PeBBX14* containing more light-responsive *cis*-elements, while *PeBBX06* and *PeBBX14* contained more hormone-responsive elements.

### Transcript expression pattern analysis

**Fig. 5:**
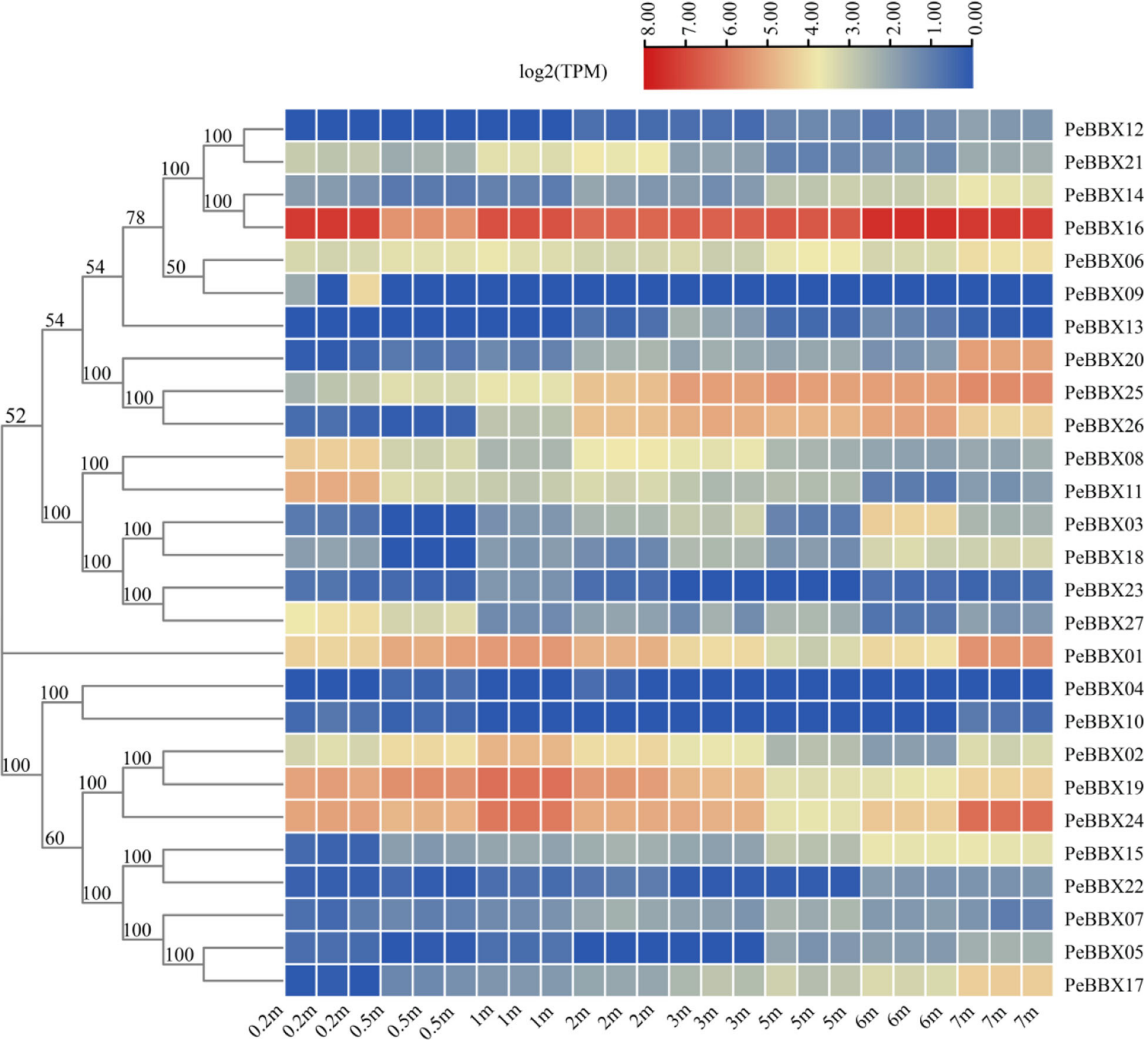
Analysis of the expression patterns of different heights of bamboo shoots. That three replicates per condition. The relative expression levels are depicted according to the colour scale, where blue indicates low abundance and red indicates high abundance. Gene expression log2 (TPM) was calculated as the sum of the abundance of all transcripts produced by a given gene

next quantified the expression patterns of the 27 PeBBX genes at different shoot heights using RNA-seq (Fig. 5, Additional file 6: Table S3). This analysis revealed that all 27 members had different expression patterns. *PeBBX16* was highly expressed ubiquitously, while *PeBBX21*, *PeBBX26*, *PeBBX08* and *PeBBX27* were highly expressed at 0.2 m. *PeBBX19* and *PeBBX24* showed a decreasing trend in expression at 4 m. *PeBBX17*, *PeBBX20* and *PeBBX25* showed a positive correlation between their expression levels and the growth height of the shoots. The gene expression results showed that the *PeBBX* genes were expressed at all stages of shoot development.

**Fig. 6:**
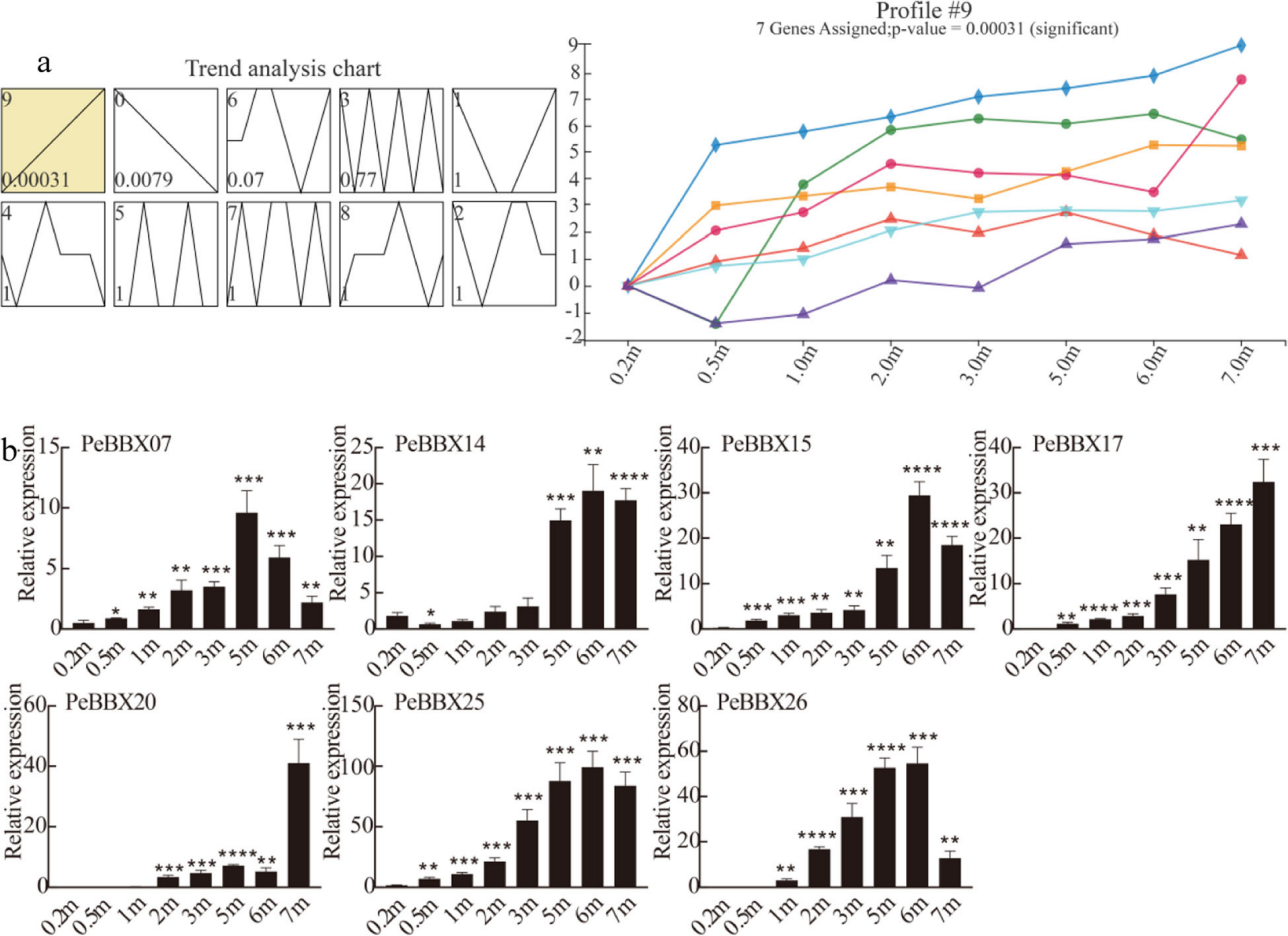
STEM analysis of *PeBBX* genes. **a** STEM analysis of *PeBBX* genes. The yellow boxes indicate significant gene expression. All 10 profiles are drawn with profile numbers denoted at the top left. The dashed line indicates the trend of expression over time, and the value in the lower-left corner is the *P*-value for its corresponding significance level. The image on the right shows the manifestations of significantly expressed genes. **b** Expression patterns of genes in profile 9 that are highly positively associated with shoot growth stages. A single asterisk indicates that the level of the gene expression was significantly different (*t*-test, *p* < 0.05). Double asterisks indicate that there is a significant difference (*t*-test, *p* < 0.01). Error bars represent standard deviations of the means of three independent replicates

STEM is a measurement of gene expression in similar samples at different time points to observe the changes in gene expression at each time point and elucidate the patterns of changes in interdependent relationships between genes. Temporal sequence analysis based on STEM was applied to the gene expression patterns of *PeBBX* genes at multiple heights during the growth of shoots, revealing 10 different gene expression profiles (Fig. 6a). Profile 9 contained 7 *PeBBX* genes, which all had expression levels that were positively correlated with shoot height. Within this profile, *PeBBX14*, *PeBBX15*, *PeBBX25* and *PeBBX26* had the highest expression at 5 m, while *PeBBX07* had the highest expression at 4 m (Fig. 6b).

### Protein interaction network

**Fig. 7:**
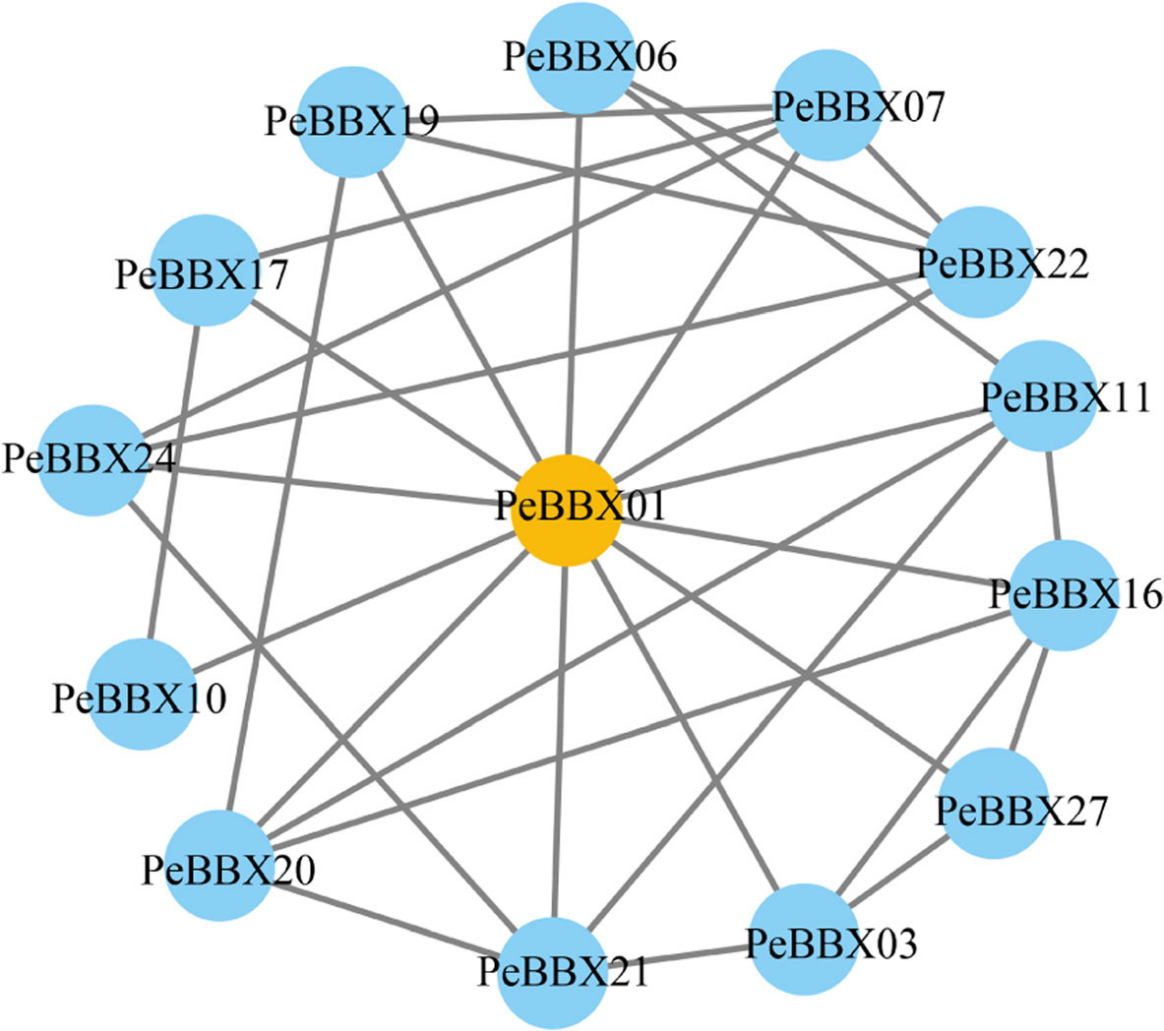
Protein interaction network analysis. Nodes indicate proteins, and edges indicate the presence of interactions between two proteins. Nodes in orange indicate the highest level of connectivity for that protein

A protein interaction network was constructed using a network modelling method at STRING, its topological properties were analyzed at the network level. This analysis revealed that 14 proteins in the BBX family were predicted to interact with each other (Fig. 7). The PeBBX01 protein had the highest connectivity, interacting with 13 BBX proteins of Moso bamboo.

### *PeBBX* target gene identification, GO, KEGG annotation and analysis.

**Fig. 8:**
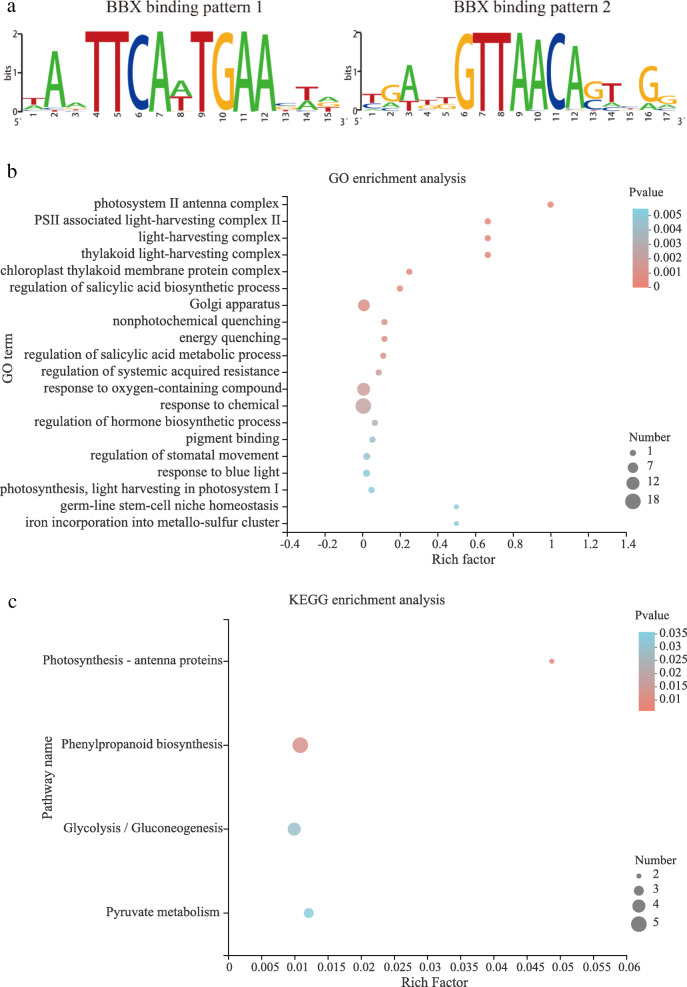
BBX binding pattern, GO and KEGG annotation. **a** BBX target gene prediction. The two predicted BBX binding patterns resulted in the targeting of two different sets of genes, termed target genes. **b** GO enrichment analysis of BBX target genes from the enrichment top 20. The vertical axis represents the GO term, and the horizontal axis represents the Rich factor, with larger Rich factor indicating greater enrichment. The size of the dots indicates the number of genes in the GO term, and the color of the dots corresponds to different *P*-value ranges. The top 20 enrichment results are displayed for *P*-value < 0.05. **c**)KEGG enrichment analysis of BBX target genes from the top 20. The vertical axis represents the pathway name, the horizontal axis represents the Rich factor and the size of the points indicates the number of genes in the pathway. The top 20 enrichment results are displayed for *P*-value < 0.05

We predicted BBX target genes using the JASPAR database and identified two BBX binding patterns (Fig. 8a), which were used to predict BBX target genes (Additional file 8). GO enrichment analysis and KEGG enrichment analysis of BBX target genes were also performed on GO enrichment tool (http://geneontology.org/page/go-enrichment-analysis) and KEGG website, respectively. GO annotation enrichment was divided into three levels: biological process (BP), cellular component (CC) and molecular function (MF). A total of 185 target genes were predicted to be regulated by BBX members and were mainly enriched in categories falling under CC and BP (Fig. 8b). At the CC level, targets were primarily enriched in the Golgi apparatus (GO:0005794), and at the BP level, targets were mainly enriched in response to oxygen-containing compound (GO:1,901,700) and response to chemical (GO:0042221). At the MF level, there was only one enriched GO term: pigment binding (GO:0031409). In addition, GO predicted that some BBX target genes were components of the photosystem complexes, including photosystem II (PSII) antenna complex, PSII associated light-harvesting complex II, light-harvesting complex and thylakoid light-harvesting complex (Additional file 9). The target genes included photopigment PR genes, such as *PH02Gene47533* and *PH02Gene42040*, both of which are Chlorophyll a-b binding proteins (CP26). These targets indicated that *PeBBX* genes were associated with the formation of complexes that is involved in the regulation of photosynthesis (Additional file 10). KEGG predicted that the major metabolic pathways enriched in BBX target genes were found to be primarily involved in Phenylpropanoid biosynthesis, Photosynthesis-antenna proteins, Pyruvate metabolism and Glycolysis/Gluconeogenesis (Fig. 8c, Additional file 11).

### Protein structure analysis by homology modeling

**Fig. 9:**
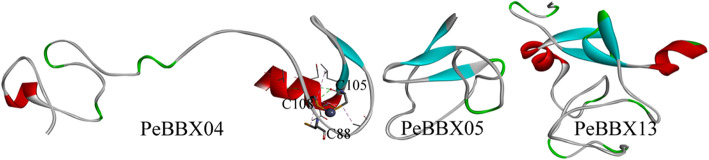
Three-dimensional structure of the Moso bamboo BBX protein sequences. Models were constructed with the SWISS-MODEL software. The red areas indicate the α-helices and the bright blue areas represent the β-folds. C88, C105 and C108 indicate cysteines bound to a zinc ion

To further analyze the protein function, the tertiary structures of the proteins were predicted. PeBBX04 from subclade I, PeBBX05 from subclade II and PeBBX13 from subclade IV were selected for tertiary structural homology modelling. The 3D structures of the three subclades were found to have different tertiary structures (Fig. 9). The PeBBX04 sequence consisted of two α-helices and two β-folded lamellae and contained three amino acid residues that specifically bind to zinc ions (C.88, C.105, C.108). The PeBBX05 sequence consisted mainly of β-folded lamellae, and the PeBBX13 sequence was more complex, with four β-folded lamellae.

### Subcellular localization of BBX proteins

**Fig. 10:**
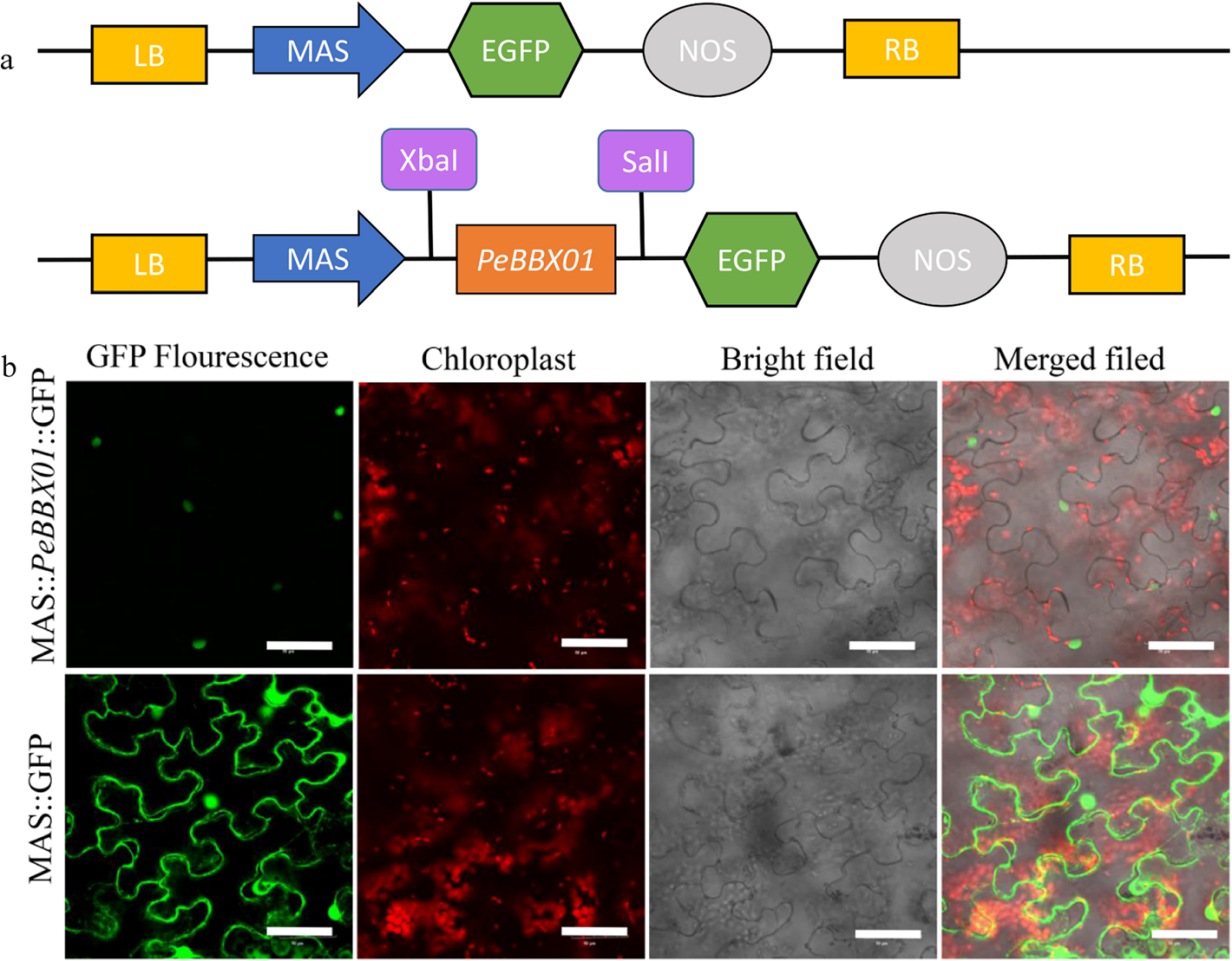
Subcellular localization of the GFP-fused PeBBX protein. **a** Schematic drawing of *PeBBX01* subcellular localization. **b** Subcellular localization of *PeBBX01*. The fusion protein MAS-*PeBBX01*-GFP and the control vector were transiently expressed in tobacco leaves and then observed by fluorescence microscopy. Scale bar was 50 μm

*PeBBX01* showed high connectivity in protein interaction network prediction, and to further understand the properties of this protein, we selected *PeBBX01* for subcellular localization analysis. We constructed a transient expression MAS-*PeBBX01*-GFP vector for this gene for validation experimental purposes. The results showed that the MAS-GFP fluorescent signal in the control was dispersed throughout the tobacco cells, and the expressed fusion protein MAS-*PeBBX01*-GFP was specifically distributed in the nucleus (Fig. 10).

## Discussion

Plants are often challenged with various unfavorable environmental conditions throughout their growth and development. In response to these environmental changes, a series of physiological and biochemical changes are produced. A diverse array of different genes are required for these changes, with TFs playing a central role [33, 34]. Zinc finger TFs are a superfamily of TFs with many members, including BBXs. In plants, several *BBX* gene families have been identified, with 32 members in *Arabidopsis*, 30 in rice, 29 in tomato, 30 in potato and 37 in pear [2, 9, 6, 35, 36].

In this study, we identified a total of 27 genes encoding BBX proteins (PeBBX) in the Moso bamboo genome. The B-box1 and B-box2 structural domains of the 27 PeBBX protein sequences were highly conserved, and there was a strong similarity with the BBX sequences of rice and *Arabidopsis*. This indicated that the sequences of BBX were strongly conserved during the evolutionary process. The number of genes encoding specific TF families varies among plant species due to evolutionary expansion and the development of species-specific functions [37]. The results of the phylogenetic tree showed that *PeBBXs* lacks the III and V subclades, and expanded the subclade IV compared to *Arabidopsis* and rice. Among the BBX family members of *Arabidopsis*, 21 BBX have been functionally characterized. Among them, there were eight BBX proteins that were members of subclade IV and had either facilitative or repressive effects on photomorphogenesis. Recent analysis of BR-related (brassinosteroid action-related) genes in bamboo has shown that the phytohormone BR regulated growth by promoting root cell division and elongation [38, 39], and *ATBBX20* in subcladeIV responded to the phytohormone BR [14]. In addition, these genes were involved in salt, cold, darkness, and hormone responses [14]. *OsBBX14*, a member of rice subclade IV. Under photoperiodic conditions, *OsBBX14* expression had a circadian rhythm and the *OsBBX14* overexpression strain (*OsBBX14*-OX) delayed heading date by repressing the expression of the flowering-forming factor gene under long-day (LD) and short-day (SD) conditions [40]. Based on studies in the subclade IV of *Arabidopsis* and rice, it is hypothesized that the expansion of the subclade IV of *PeBBX* may have an effect on flowering and photomorphogenesis and development phenomena in Moso bamboo.

The motif analysis revealed that different BBX proteins in subclades I, II and IV contain motifs with unclear functions. In addition, there were three BBX proteins (PeBBX02, PeBBX19, PeBBX24) had a valine-proline (VP) motif consisting of 6 amino acids at the C-terminus, with a consistent sequence of G-I/V-V-V-P-S/T-F (Additional file 3: Figure S3). The VP motif is generally about 16–20 amino acid residues away from the CCT structural domain, which plays a vital role in the interaction between BBX protein and coiled-coil proteins [41, 42]. COP1 (CONSTITUTIVE PHOTOMORPHOGENIC 1) is involved in the degradation of HY5 as a pan-peptide ligase component and inhibits plant photomorphogenesis [43]. Additionally, it was previously shown that different VP motifs allow different BBXs to bind the WD40 (Trp-Asp-40) structural domain of COP1 with different affinities, thereby regulating photomorphogenesis [44].

The amino acid sequences of B-box1 and B-box2 structural domains in animals is significantly different compared to plants, but within plants these domains are much more consistent. In addition, the evolutionary trajectory of BBX domains in green plants indicates that the original BBX protein had only one BBX domain, which underwent a duplication event later in evolution. After this event, the CCT domain was formed. The deletion of the 2 BBX and CCT structural domains during the subsequent evolution, as well as the further replication events of the BBX structural domain, helped BBX proteins expand into different structural types [7]. PeBBX09 had only one BOX1 domain, presumably due to a deletion that occurred during evolution.

Gene duplication has been shown to be a significant factor in the evolution of genes with novel functions [45]. In the process of species evolution, the duplication events can promote the evolution of species and the rapid expansion of the species genome, and most of the duplication events occur in species due to the drastic changes in the survival environment [46]. Furthermore, during the growth and development of species, gene duplication events mainly help species to adapt to different environments, thus adapting to the drastically changing external environment and increasing the survival rate of the species [47, 48]. Jiao et al. [49] showed that one of the gene duplication events in plants occurred 192 mya. In addition, it has been reported that the Moso bamboo *SPL* gene family had a large duplication event at about 15 mya [26]. Among the gene pairs identified, *PeBBX14*/*PeBBX16*, *PeBBX17*/*PeBBX05*, *PeBBX18*/*PeBBX03*, *PeBBX19*/*PeBBX02*, *PeBBX21*/*PeBBX12*, *PeBBX23*/*PeBBX27*, *PeBBX06*/*PeBBX09* and *PeBBX08*/*PeBBX11* occurred between 9 and 15 mya, close to the previously reported large-scale duplication events of 7–15 mya for the whole genome of Mao bamboo [26].

In gene family evolution and expansion, tandem duplication and segmental duplication occur frequently [46]. Zhang et al.[50] found that fragment duplication was the main driving force for the expansion of the RPD3 gene family in cotton in a covariance analysis. In the Moso bamboo *BBX* gene family, no tandem gene clusters were identified, and 24 gene pairs were identified, suggesting that segmental duplication events may be more important than tandem duplication in the expansion of the *PeBBX* genes. Interestingly, two genes of these homologous gene pairs belonged to the same subclade, suggesting that some BBX family genes might arise from gene duplication events and that these duplication events were the main drivers of gene family expansion [51]. As a result of interspecies collinearity analysis, some homologous regions were formed between different chromosomes. Some *PeBBX* genes fell within these homologous regions, suggesting that genome-wide amplification of the *BBX* gene family also occurred. The expansion of BBX during evolution and their high conservation throughout different plant species suggest that BBX may play an essential role in the adaptation of plants to terrestrial environments [52, 14].

Plant hormones were originally described as regulators during growth and development, acting as signaling molecules to regulate complex metabolic pathways [53, 54]. In a previously study, four tomato BBX genes were induced by ethylene (ETH) and all of them had ERE in their promoters [2]. In grapes, four BBX genes expression (*VvBBX2*, *VvBBX3*, *VvBBX13* and *VvBBX22*) were affected by ABA and ETH, and the promoter regions of all four genes contained ABRE promoter elements [55]. Three *PhBBX* genes (*PhBBX8*, *PhBBX15* and *PhBBX20*) contained drought-responsive *cis*-elements were affected by drought in *Petunia hybrida* [56]. Moso bamboo has the property of explosive growth, which can grow about 20 m in 1.5 months [57]. It was found that the growth of Moso bamboo was correlated with GA. Exogenous gibberellin (GA3) was applied to Moso bamboo seedlings and it was observed to significantly increase internode length [58]. In the currently study, there were 9 genes containing ERE promoter elements and 14 genes contained drought response elements (MBS), and almost all of them contained ABRE. In addition, a large number of light response elements, hormone response elements and abiotic stress response elements were distributed in the promoters of *PeBBX*s, suggesting that these genes might play essential functions in light signaling, hormone and stress response.

There were studies have shown that growth regulation effect of BBX [59]. *AtBBX24* binded to DELLA protein and prevented DELLA-mediated inhibition of *PIF4* activity, which in turn *PIF4* binded to the promoter of cell elongation-related genes to promote cell elongation [60]. It has been shown that expression of apple *MdBBX10* in *Escherichia coli* enhanced cellular tolerance to salt and osmotic stress, and overexpression of this gene in *Arabidopsis* also enhanced the tolerance of transgenic plants to abiotic stresses, such as drought and salt [61, 62]. High salt and polyethylene glycol (PEG) treatment-induced *SsBBX24* gene expression and protein accumulation in potato (*Solanum sogarandinum*), and the length of daylight also regulated the response of *SsBBX24* to salt stress [63]. In addition to direct regulation at the transcriptional and protein levels, BBX proteins have been shown to play important roles in hormone signaling by growth hormones, such as indole-3-acetic acid (IAA), gibberellic acid (GA), ABA and brassinolide (brassinosteroid, BR) [64]. *AtBBX21* acts as a negative regulator related to growth hormones, ETH and BR, thus affecting plant growth under long-term shade conditions [65]. Additionally, GA may be involved in the regulation of flowering in *Dendranthema morifolium* by *CmBBX24* [22]. Additionally, our expression analysis indicated that *PeBBX* genes might play an important role in the process of plant growth and development, different *PeBBX* genes had expression specificity at different heights.

Protein interaction networks contribute to the understanding of complex biological network systems [66]. Recent studies have revealed that the plant BBX structural domain plays an important role in mediating protein interactions and regulation of gene expression. In plants, the BBX domain functions by forming heterodimers within the BBX protein family. In *Arabidopsis*, *AtBBX24* and *AtBBX25* have been shown to interfere with the function of HY5 (ELONGATED HYPOCOTYL 5) by forming an inactive heterodimeric form, which in turn affected *AtBBX22*/*LZF1* (LIGHT-REGULATED ZINC FINGER PROTEIN 1) expression and inhibited the photomorphogenesis of seedlings [10]. *Arabidopsis* B-BOX32 interacts with BBX4/CONSTANS-LIKE3 (COL3) to regulate flowering [67].

In addition, BBX proteins from different plants can also have cross-species effects. For example, the N-terminal BBX region of *AtBBX32* in *Arabidopsis* has been shown to interact with the BBX protein of *GmBBX62* in soybean (*Glycine max*) [12]. Protein interaction network analysis speculated that PeBBX proteins interact and PeBBX01 interacted with multiple proteins and it might play important roles within the Moso bamboo family.

BBX, as a transcription factor, can regulate other proteins and thus affect plant growth and development. For example, *CmBBX8* accelerated flowering in chrysanthemums by directly targeting *CmFLT1*, a flower-inducible gene [68]. *BBX16* promoted the growth of shade hypocoty possibly as a positive transcriptional regulator of *PIL1* [65]. Enrichment analysis of the top 20 BBX target genes predicted that many were involved in the photosynthetic pathway, such as the PSII antenna complex, PSII associated light-harvesting complex II, light-harvesting complex and others. It has been found that PSII is the first complex involved in oxygenic photosynthesis [69]. PSII is a multisubunit pigment-protein complex that catalyzes light-driven water oxidation and reduction of plastid quinones. The light-trapping chlorophyll a/b binding protein is the light-carrying protein of the PS II light-trapping complex. Photons captured by the light-trapping chlorophyll a/b-binding antenna complex light-catching complex II (LHCII) are converted by photosynthesis into biochemical energy and biomass for regulating plant growth, development and morphogenesis [70–73].

The phenylpropanoid/flavonoid biosynthesis pathway is an important metabolic pathway in plants. For example, plant defense responses are associated with activation of the general phenylpropanoid pathway and anthocyanins are the end product of a branch of the phenylpropanoid/flavonoid biosynthesis pathway [74, 75]. It has been shown that BBX proteins not only play an essential role in regulating photomorphogenesis [76], but also light-induced anthocyanin synthesis. It was previously demonstrated that in *Arabidopsis*, HY5 is a core regulator that facilitates the photomorphogenesis process and that *AtBBX32* can bind to *AtBBX21* and then interact with HY5 to reduce its transcriptional activity [77, 78]. In contrast, *AtBBX24* might interfere with the binding of HY5 to the promoter of anthocyanin genes by forming a heterodimer with HY5 [79]. *PpBBX16* in pear (*Pyrus pyrifolia*) has also been shown to be a positive regulator of anthocyanin in berries [80]. The enrichment analysis of target genes of BBX were also closely related to the carbohydrate metabolism pathway. It has been described previously that carbohydrates play an important role in plant growth and stress tolerance [81, 82]. Overall, the analysis of the KEGG metabolic pathway led to a better understanding of the target genes and the pathways regulated by *PeBBX*.

## Conclusions

In this study, 27 members of the Moso bamboo BBX family were examined and divided into three subclades based on conserved domains. Some fragment duplications were found on different chromosomes, which likely drove the expansion of the *PeBBX* gene family. Bioinformatic analysis of gene structure, conserved motifs, chromosomal position, covariance and *cis*-element prediction greatly expanded the current understanding of the structural relationships among family members. Target gene prediction of *PeBBX* genes resulted in the identification of several key functional genes, opening up a number of new avenues for exploring the role of these genes in Moso bamboo development.

## Materials and methods

### Identification of *BBX* genes in the *P. edulis* genome

The Moso bamboo genome was downloaded from GigaDB (http://gigadb.org/dataset/100498). In order to identify the BBX TF genes in *P. edulis*, the BBX domain Hidden Markov Model (HMM) was used as a query to search the Moso bamboo genome. After removing redundant hits and initial chromosomal localization, BBX family genes were identified by further analysis using CDD (http://www.ncbi.nlm.nih.gov/Structure/cdd/wrpsb.cgi) to reveal potential non-redundant BBX TF genes, termed *PeBBX* genes. The Molecular weight (MW) and isoelectric point (pI) of the deduced amino acid sequences were predicted with the ExPASy online tool. Next, the SignalP 4.1 Server (http://www.cbs.dtu.dk/services/SignalP/) was used to predict signal peptides of each member of the BBX family [83, 84]. Finally, Plant-mPLoc was employed to predict the subcellular localization of proteins [85].

### Phylogenetic analysis

The BBX protein sequences of *Arabidopsis* and rice were downloaded from the Arabidopsis Information Resource, version 10 (TAIR 10) (http://www.arabidopsis.org) and the RGAP 7 database (Rice Genome Annotation Project, http://rice.plantbiology.msu.edu/). The BBX protein sequences from *A. thaliana*, *Oryza sativa* and *P. edulis* were used to construct a phylogenetic tree using the neighbor-joining (NJ) method. For statistical reliability, the nodes of the tree were assessed by bootstrap analysis containing 1000 replicates.

### Analysis of gene structure, motifs, domains and multi-sequence alignments

To better understand the structures of the *PeBBX* genes, their exon-intron information was obtained from the whole genome GFF annotation file, then visualized with TBtools. To better characterize the structures of the PeBBX proteins, the software Multiple Expectation Maximization for Motif Elicitation (MEME) was used to identify conserved motifs [86]. The following optimization parameters were used: any number of repeats, optimum width of each motif restricted between 6 and 50 residues and a maximum number of 5 motifs. Alignments of protein sequences and the corresponding residues were carried out using Geneious software with embedded parameters set to cost matrix: Blosum62. In addition, conserved domain analysis was performed using Conserved Domain Search (https://www.ncbi.nlm.nih.gov/Structure/cdd/wrpsb.cgi).

### *Cis*-acting elements in the *PeBBX* gene promoter regions

In order to identify conserved *cis*-elements within the promoter regions of the *PeBBX* genes, the sequences 1500 bp upstream of the initiation codons were considered as the proximal promoter region sequences. These sequences were submitted to the PlantCARE database for promoter prediction analysis (http://bioinformatics.psb.ugent.be/webtools/plantcare/html/), followed by functional categorization and visualization using TBtools [87].

### Chromosomal locations, genomic duplications and Ka/Ks ratios

To identify the locations of the *PeBBX* members on the Moso bamboo chromosomes, the termination and start positions of the coding sequences (CDSs) were retrieved from the Moso bamboo database. Each *PeBBX* gene was placed on the corresponding Moso bamboo chromosome according to the physical location of the gene and schematically mapped through the MapChart online website. Circos software was used to visualize the gene duplications of Moso bamboo *PeBBX* genes [88]. Sequences from rice, *Arabidopsis*, pepper and maize were identified by BLAST comparison to determine homologous gene pairs [89]. Then, MCScanX was used to identify homologous regions [90].

The ratio of non-synonymous substitutions rate (Ka)/synonymous substitutions rate (Ks) was used to determine the homologous relationship and divergence time of *PeBBX* genes. For Ka/Ks analysis, 24 homologous gene pairs were identified by BLAST. Ka/Ks ratios between homologous gene pairs were calculated using KaKs_Calculator 2.0 [91]. The evolutionary divergence time within the*PeBBX* genes was computed using the bamboo-specific divergence time formula T = Ks/2λ (λ = 6.5 × 10^− 9^) [24].

### Expression patterns of *PeBBX* genes

To analyze the expression patterns of the *PeBBX* genes and identify genes that may be related to rapid shoot development, we downloaded RNA-seq data (Accession: SRX3282084, SRX3282085, SRX3282086, SRX3282087, SRX3282088, SRX3282089, SRX3282090, SRX3282091, SRX3282092, SRX3282093, SRX3282094, SRX3282095, SRX3282096, SRX3282097, SRX3282098, SRX3282099, SRX3282100, SRX3282101, SRX3282102, SRX3282103, SRX3282104, SRX3282105, SRX3282106, SRX3282107) from the gene expression profile database of NCBI (http://www.ncbi.nlm.nih.gov/geo/). Transcriptomic data with replicates came from bamboo shoots at different growth stages (0.2 m, 0.5 m, 1 m, 2 m, 3 m, 5 m, 6 and 7 m). Transcriptome data which was quantified as transcripts per million reads (TPM) were log2-transformed [92] and imported into TBtools, where Amazing Heatmap was used to generate expression heatmaps. For time-series gene expression analysis, we further employed the STEM algorithm with a number of time-series patterns of 10 and a significant trend P-value set to 0.001.

### Protein-protein interaction (PPI) network construction

PPI relationships were analyzed using protein sequences from PeBBX members. Prediction of PPI networks was performed using the Retrieval of Interacting Genes/Proteins online database (STRING v10) (http://string-db.org) [93]. Cytoscape is an open-source bioinformatics software platform used for visualizing molecular interaction networks [94], which we employed to build PPI network maps. The minimum required score for the interaction was set to ≥ 0.15 when constructing the interaction network.

### Plant Material, RNA extraction and quantitative real-time (qRT-PCR) analysis

In order to analyze the tissue expression pattern of *PeBBX* genes, various growth stages samples of Moso bamboo tissues were collected from bamboo plants growing in the local Moso bamboo forests in Guilin, Guangxi (All samples collection with protocols approved by the owner), including various young parts of 0.2 m, 0.5 m, 1 m, 2 m, 3 m, 5 m, 6 and 7 m. The collected samples were kept in three independent replicates and all samples were stored at -80 °C until further experiments, followed by grinding to fine powder with a mortar and pestle.

A FastPure Plant Complete RNA Isolation Package was utilized to extract RNA from Moso bamboo samples for qRT-PCR analysis, following the manufacturer’s instructions (Vazyme company, China). First-strand cDNA was synthesized with a HiScript@ lll 1st Strand cDNA Synthesis Kit (+ gDNA wiper), following the manufacturer’s instruction. Gene-specific qRT-PCR primers were designed by Primer Premier 5 (See Additional file 7: Table S4) based on the CDS of each gene. Each qRT-PCR experiment was conducted in triplicate using separate RNA samples. The reaction conditions were: 95 °C for 5 min, followed by 38 cycles of 95 °C for 15 s and 55 °C for 15 s. A melting curve from 65 to 95 °C was then used. The *actin* gene was used as an internal control for normalization. Data were subjected to one-way analysis of variance (ANOVA), followed by Tukey’s test using SPSS software [95].

### *PeBBX* target gene identification, annotation and expression analysis

Gene promoter sequences (2000 bp upstream of the start site) were extracted from the genome of Moso bamboo. The JASPAR database (http://jaspar.genereg.net/) was used to predict the binding site genes of *PeBBX* [96]. The screening index was set to less than 1.0 E^− 6^ to identify the genes targeted by BBX members.

Geno ontology (GO) annotation was performed using the UniProt-GOA database (www.http://www.ebi.ac.uk/GOA/). Protein IDs were converted to UniProt IDs for GO mapping analysis and KEGG protein pathway annotation based on the KEGG database (http://www.kegg.jp/kegg/ko.html) [97].

### Protein structure analysis by homology modeling

The three-dimensional (3D) structures of PeBBX proteins were predicted in order to analyze potential protein functions. The tertiary structures of PeBBX proteins were generated by homology modeling techniques using the online template-based SWISS-MODEL (http://swissmodel.expasy.org) [98].

### Subcellular localization of BBX proteins

To verify the localization of *PeBBX01* in plant cells, the *PeBBX01* gene was selected to design primers with a homologous recombination arm (the primers see Additional file 12), and the full-length CDS sequence with no terminator codon amplified from the cDNAs of Moso bamboo, then the fragment was cloned into the p1300-GFP vector. Within this vector, the *PeBBX01* gene was fused with the green fluorescent protein (GFP) gene under the control of the promoter to form a recombinant vector. The accuracy of the positive clone was ensured by PCR and DNA sequencing, and it was transformed into the *Agrobacterium tumefaciens* strain GV3101 [99, 100],. Tobaccos (*Nicotiana benthamiana*) were cultivated in a greenhouse at 23 °C for about one month, then the tobacco in good growth condition was selected, and *Agrobacterium tumefaciens* carrying the recombinant vector was injected into the third or fourth leaf and incubated at 25 °C for 24 h in the dark, followed by 24–72 h in the light, to make sections for observation under a laser confocal microscope (*Olympus, Tokyo, Japan*) [55]

## Supplementary Information

**Additional file 1: Figure S1.** The distribution of the *PeBBX*genes on the scaffolds of Moso bamboo. *PeBBX* genes are numbered 1-27. The chromosome number is shown at the top of each strip, with the gene name displayed on the right or left side of the chromosome.

**Additional file 2: Figure S2.** Location of *cis*-elements in the promoters of *PeBBX* genes. *Cis*-element analysis of *PeBBX*s. The 1500 bp DNA fragments upstream of the ATG were analyzed using the online analysis software PlantCARE. Different *cis*-acting elements of *PeBBX*genes are displayed. The different colored markers indicate different predicted*cis*-acting elements.

**Additional file 3: Figure S3.** VP motif. Valine-proline (VP) motif, the proteins with red underlines possess typical VP residues.

**Additional file 4: Table S1. **The IDs and sequences of BBX proteins from rice, *Arabidopsis*and Moso bamboo used in phylogenetic tree construction.

**Additional file 5: Table S2.** Cis-element analysis of PeBBX genes.

**Additional file 6: Table S3.** The FPKM value of PeBBX genes in different heights.

**Additional file 7: Table S4. **The primers used for qRT-PCR.

**Additional file 8.** BBX target genes

**Additional file 9:** The founctional GO analysis.

**Additional file 10:** The founctional annotation of BBX target genes.

**Additional file 11:** The founctional KEGG analysis.

**Additional file 12: ** The primers of PeBBX01.

## Data Availability

All data supporting the conclusions of this article are provided within the article and its additional files. The genomics sequences data of Moso bamboo and rice are available in the GigaDB Database (http://gigadb.org/dataset/100498), the Arabidopsis Information Resource, version 10 (TAIR 10) (http://www.arabidopsis.org) and the RGAP 7 database (Rice Genome Annotation Project, http://rice.plantbiology.msu.edu/). The gene expression profile database of NCBI (http://www.ncbi.nlm.nih.gov/geo/), accession: SRX3282084, SRX3282085, SRX3282086, SRX3282087, SRX3282088, SRX3282089, SRX3282090, SRX3282091, SRX3282092, SRX3282093, SRX3282094, SRX3282095, SRX3282096, SRX3282097, SRX3282098, SRX3282099, SRX3282100, SRX3282101, SRX3282102, SRX3282103, SRX3282104, SRX3282105, SRX3282106, SRX3282107.

## Abbreviations

TFs: Transcription factors
BBX: B-box
CCT: CONSTANS, CCMike and TOC
BBX 1: B1
BBX 2: B2
CO: CONSTAVS
FT: FLOWERING LOCUS T
MeJA: Methyl jasmonate
ABA: Abscisic acid
ARE: Anaerobic response regulatory elements
LTR: Low-temperature response elements
BP: Biological process
CC: Cellular component
MF: Molecular function
PSII: Photosystem II
VP: Valine-proline
COP1: CONSTITUTIVE PHOTOMORPHOGENIC 1
PEG: Polyethylene glycol
IAA: Indole-3-acetic acid
GA: Gibberellic acid
BR: Brassinolide (brassinosteroid)
HY5: ELONGATED HYPOCOTYL 5
LZF1: LIGHT-REGULATED ZINC FINGER PROTEIN 1
LHCII: Light-catching complex II
MEME: Multiple Expectation Maximization for Motif Elicitation
CDS: Coding sequences
TPM: Transcripts per million reads
PPI: Protein-protein interaction
qRT-PCR: Quantitative real-time
STEM: Short time-series expression miner
LD: Long-day
SD: Short-day
ETH: Ethylene
GFP: Green fluorescent protein
