# Supplementary material for: The BBX gene family in Moso bamboo (Phyllostachys edulis): identification, characterization and expression profiles
Source: BMC Genomics. 2021 Jul 13;22:533. doi: 10.1186/s12864-021-07821-w (PMC8276415; doi:10.1186/s12864-021-07821-w)
Supplement: Supplementary file 1 — Additional file 1: Figure S1. The distribution of the PeBBXgenes on the scaffolds of Moso bamboo. PeBBX genes are numbered 1-27. The chromosome number is shown at the top of each strip, with the gene name displayed on the right or left side of the chromosome. [file 12864_2021_7821_MOESM1_ESM.docx]

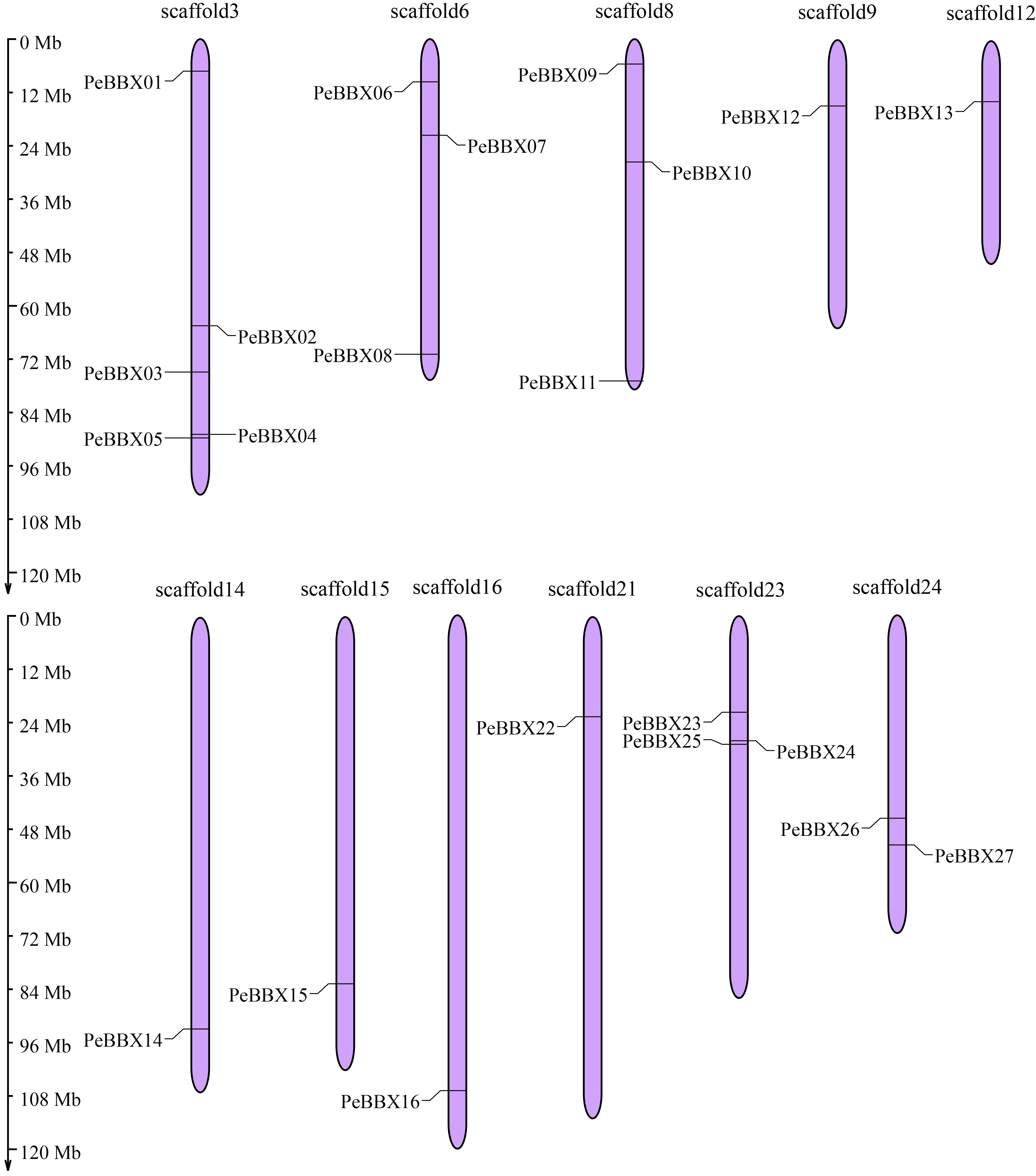


Figure S1 The distribution of the *PeBBX* genes on the scaffolds of Moso bamboo.

*PeBBX* genes are numbered 1-27. The chromosome number is shown at the top of each strip, with the gene name displayed on the right or left side of the chromosome.
