# Supplementary material for: The BBX gene family in Moso bamboo (Phyllostachys edulis): identification, characterization and expression profiles
Source: BMC Genomics. 2021 Jul 13;22:533. doi: 10.1186/s12864-021-07821-w (PMC8276415; doi:10.1186/s12864-021-07821-w)
Supplement: Supplementary file 2 — Additional file 2: Figure S2. Location of cis-elements in the promoters of PeBBX genes. Cis-element analysis of PeBBXs. The 1500 bp DNA fragments upstream of the ATG were analyzed using the online analysis software PlantCARE. Different cis-acting elements of PeBBXgenes are displayed. The different colored markers indicate different predictedcis-acting elements. [file 12864_2021_7821_MOESM2_ESM.docx]

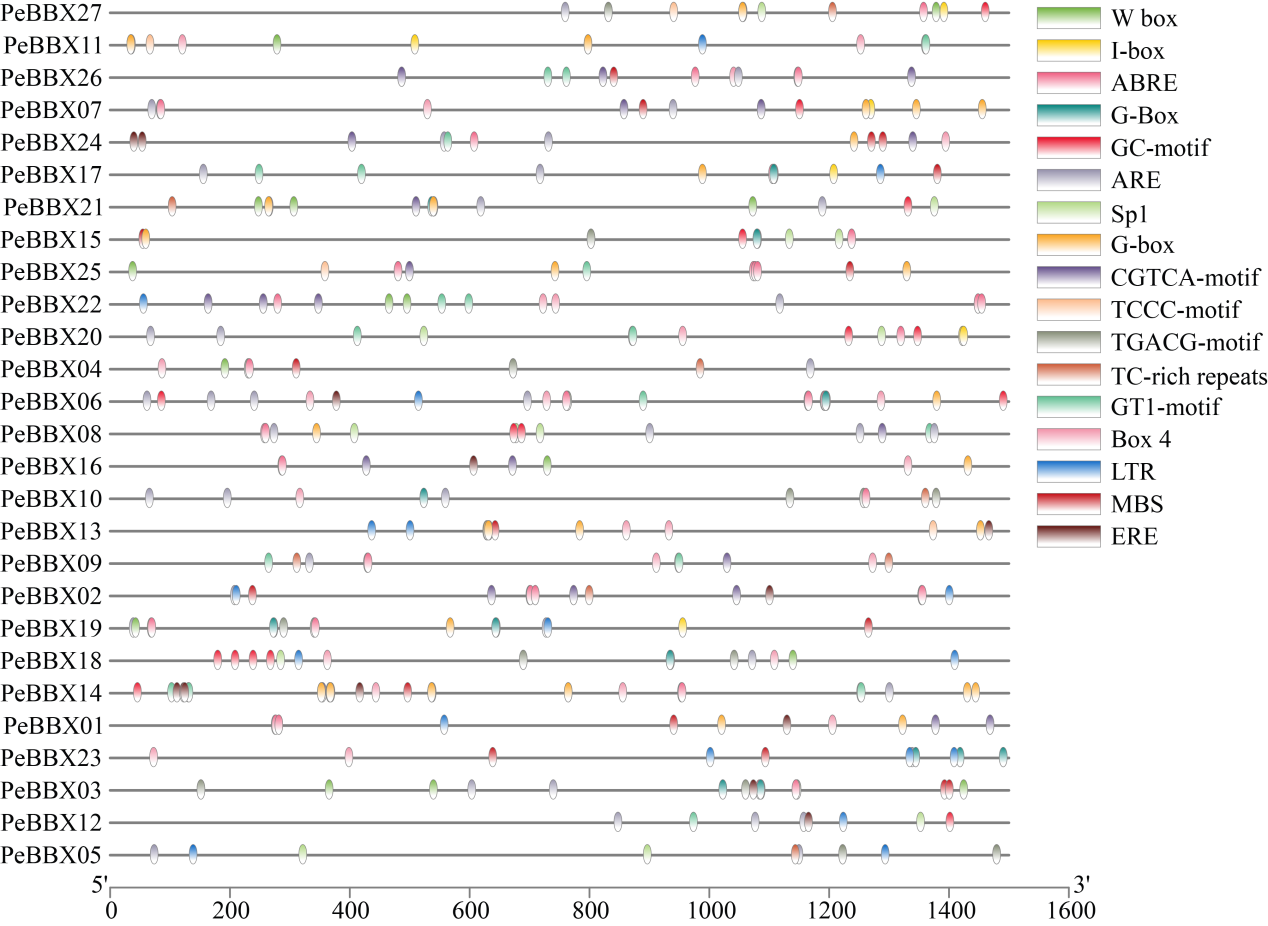


Figure S2: Location of *cis*-elements in the promoters of *PeBBX* genes.

*Cis*-element analysis of *PeBBX*s. The 1500 bp DNA fragments upstream of the ATG were analyzed using the online analysis software PlantCARE. Different *cis*-acting elements of *PeBBX* genes are displayed. The different colored markers indicate different predicted *cis*-acting elements.
