# Supplementary material for: The BBX gene family in Moso bamboo (Phyllostachys edulis): identification, characterization and expression profiles
Source: BMC Genomics. 2021 Jul 13;22:533. doi: 10.1186/s12864-021-07821-w (PMC8276415; doi:10.1186/s12864-021-07821-w)
Supplement: Supplementary file 3 — Additional file 3: Figure S3. VP motif. Valine-proline (VP) motif, the proteins with red underlines possess typical VP residues. [file 12864_2021_7821_MOESM3_ESM.docx]

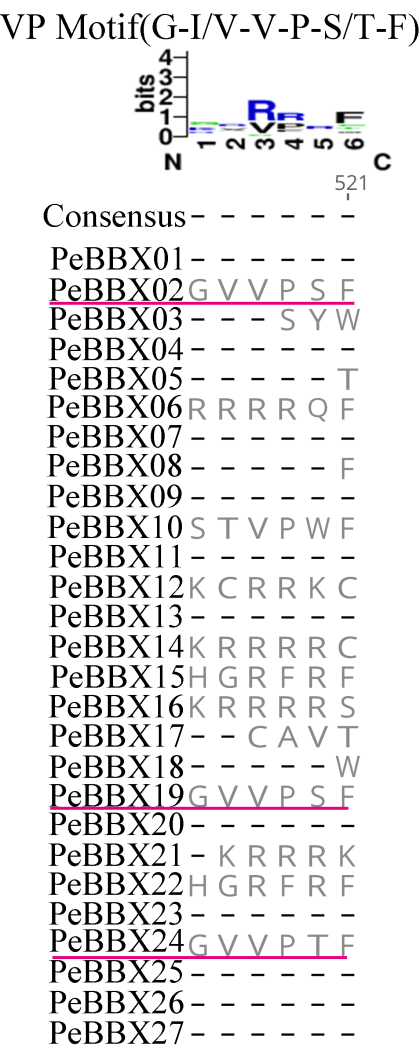


Figure S3: VP motif.

Valine-proline (VP) motif, the proteins with red underlines possess  [typical](C:/Users/Administrator/AppData/Local/youdao/dict/Application/8.9.6.0/resultui/html/index.html#/javascript:;) VP residues.
