# Supplementary material for: The BBX gene family in Moso bamboo (Phyllostachys edulis): identification, characterization and expression profiles
Source: BMC Genomics. 2021 Jul 13;22:533. doi: 10.1186/s12864-021-07821-w (PMC8276415; doi:10.1186/s12864-021-07821-w)
Supplement: Supplementary file 4 — Additional file 4: Table S1. The IDs and sequences of BBX proteins from rice, Arabidopsisand Moso bamboo used in phylogenetic tree construction. [file 12864_2021_7821_MOESM4_ESM.docx]

**Table S1. The IDs and sequences of BBX proteins from rice, *[Arabidopsis](file:///C:/Users/Administrator/AppData/Local/youdao/dict/Application/8.9.6.0/resultui/html/index.html#/javascript:;)* and Moso bamboo used in phylogenetic tree construction.**

| **ID** | **ID Rename** | **Sequences** |
| --- | --- | --- |
| AT1G06040.1 | AtBBX24 | MKIQCDVCEKAPATVICCADEAALCPQCDIEIHAANKLASKHQRLHLNSLSTKFPRCDICQEKAAFIFCVEDRALLCRDCDESIHVANSRSANHQRFLATGIKVALTSTICSKEIEKNQPEPSNNQQKANQIPAKSTSQQQQQPSSATPLPWAVDDFFHFSDIESTDKKGQLDLGAGELDWFSDMGFFGDQINDKALPAAEVPELSVSHLGHVHSYKPMKSNVSHKKPRFETRYDDDDEEHFIVPDLG |
| AT1G25440.1 | AtBBX15 | MMKSLANAVGAKTARACDSCVKRRARWYCAADDAFLCQSCDSLVHSANPLARRHERVRLKTASPAVVKHSNHSSASPPHEVATWHHGFTRKARTPRGSGKKNNSSIFHDLVPDISIEDQTDNYELEEQLICQVPVLDPLVSEQFLNDVVEPKIEFPMIRSGLMIEEEEDNAESCLNGFFPTDMELEEFAADVETLLGRGLDTESYAMEELGLSNSEMFKIEKDEIEEEVEEIKAMSMDIFDDDRKDVDGTVPFELSFDYESSHKTSEEEVMKNVESSGECVVKVKEEEHKNVLMLRLNYDSVISTWGGQGPPWSSGEPPERDMDISGWPAFSMVENGGESTHQKQYVGGCLPSSGFGDGGREARVSRYREKRRTRLFSKKIRYEVRKLNAEKRPRMKGRFVKRASLAAAASPLGVNY |
| AT1G28050.1 | AtBBX13 | MSSSERVPCDFCGERTAVLFCRADTAKLCLPCDQQVHTANLLSRKHVRSQICDNCGNEPVSVRCFTDNLILCQECDWDVHGSCSVSDAHVRSAVEGFSGCPSALELAALWGLDLEQGRKDEENQVPMMAMMMDNFGMQLDSWVLGSNELIVPSDTTFKKRGSCGSSCGRYKQVLCKQLEELLKSGVVGGDGDDGDRDRDCDREGACDGDGDGEAGEGLMVPEMSERLKWSRDVEEINGGGGGGVNQQWNATTTNPSGGQSSQIWDFNLGQSRGPEDTSRVEAAYVGKGAASSFTINNFVDHMNETCSTNVKGVKEIKKDDYKRSTSGQVQPTKSESNNRPITFGSEKGSNSSSDLHFTEHIAGTSCKTTRLVATKADLERLAQNRGDAMQRYKEKRKTRRYDKTIRYESRKARADTRLRVRGRFVKASEAPYP |
| AT1G49130.1 | AtBBX17 | MTSHQNIKISEKIMISKYQEDVKQPRACELCLNKHAVWYCASDDAFLCHVCDESVHSANHVATKHERVCLRTNEISNDVRGGTTLTSVWHSGFRRKARTPRSRYEKKPQQKIDDERRREDPRVPEIGGEVMFFIPEANDDDMTSLVPEFEGFTEMGFFLSNHNGTEETTKQFNFEEEADTMEDLYYNGEEEDKTDGAEACPGQYLMSCKKDYDNVITVSEKTEEIEDCYENNARHRLNYENVIAAWDKQESPRDVKNNTSSFQLVPPGIEEKRVRSEREARVWRYRDKRKNRLFEKKIRYEVRKVNADKRPRMKGRFVRRSLAIDS |
| AT1G60250.1 | AtBBX26 | MAQVCHTCRHVTAVIHCVTEALNFCLTCDNLRHHNNIHAEHVRYQLCDNCSMYPSILFCYEDGMVLCQSCYSHHYNCATNGHQTQVVFANMNNQHHDHAHMPHVVHHNNNNNHQQQHVGGHQRRAEMFERSCHGDNNCERWMFAMRCELCVASNSNAVVYCPTHNQILCDSCDRMIHSHEDAVPPHSRCKLCVICKRPSRRFLIGGYQFNFPPVHPPAAEGIPVTPPTELPQQDINYDYLDDVDDFSWFGR |
| AT1G68520.1 | AtBBX14 | MMKSLASAVGGKTARACDSCVKRRARWYCAADDAFLCHACDGSVHSANPLARRHERVRLKSASAGKYRHASPPHQATWHQGFTRKARTPRGGKKSHTMVFHDLVPEMSTEDQAESYEVEEQLIFEVPVMNSMVEEQCFNQSLEKQNEFPMMPLSFKSSDEEDDDNAESCLNGLFPTDMELAQFTADVETLLGGGDREFHSIEELGLGEMLKIEKEEVEEEGVVTREVHDQDEGDETSPFEISFDYEYTHKTTFDEGEEDEKEDVMKNVMEMGVNEMSGGIKEEKKEKALMLRLDYESVISTWGGQGIPWTARVPSEIDLDMVCFPTHTMGESGAEAHHHNHFRGLGLHLGDAGDGGREARVSRYREKRRTRLFSKKIRYEVRKLNAEKRPRMKGRFVKRSSIGVAH |
| AT1G73870.1 | AtBBX16 | MVVDVESRTASVTGEKMAARGCDACMKRSRASWYCPADDAFLCQSCDASIHSANHLAKRHERVRLQSSSPTETADKTTSVWYEGFRRKARTPRSKSCAFEKLLQIESNDPLVPELGGDEDDGFFSFSSVEETEESLNCCVPVFDPFSDMLIDDINGFCLVPDEVNNTTTNGELGEVEKAIMDDEGFMGFVPLDMDLEDLTMDVESLLEEEQLCLGFKEPNDVGVIKEENKVGFEINCKDLKRVKDEDEEEEEAKCENGGSKDSDREASNDKDRKTSLFLRLDYGAVISAWDNHGSPWKTGIKPECMLGGNTCLPHVVGGYEKLMSSDGSVTRQQGRDGGGSDGEREARVLRYKEKRRTRLFSKKIRYEVRKLNAEQRPRIKGRFVKRTSLLT |
| AT1G75540.1 | AtBBX21 | MKIRCDVCDKEEASVFCTADEASLCGGCDHQVHHANKLASKHLRFSLLYPSSSNTSSPLCDICQDKKALLFCQQDRAILCKDCDSSIHAANEHTKKHDRFLLTGVKLSATSSVYKPTSKSSSSSSSNQDFSVPGSSISNPPPLKKPLSAPPQSNKIQPFSKINGGDASVNQWGSTSTISEYLMDTLPGWHVEDFLDSSLPTYGFSKSGDDDGVLPYMEPEDDNNTKRNNNNNNNNNNNTVSLPSKNLGIWVPQIPQTLPSSYPNQYFSQDNNIQFGMYNKETSPEVVSFAPIQNMKQQGQNNKRWYDDGGFTVPQITPPPLSSNKKFRSFW |
| AT1G78600.1 | AtBBX22 | MKIQCNVCEAAEATVLCCADEAALCWACDEKIHAANKLAGKHQRVPLSASASSIPKCDICQEASGFFFCLQDRALLCRKCDVAIHTVNPHVSAHQRFLLTGIKVGLESIDTGPSTKSSPTNDDKTMETKPFVQSIPEPQKMAFDHHHHQQQQEQQEGVIPGTKVNDQTSTKLPLVSSGSTTGSIPQWQIEEIFGLTDFDQSYEYMENNGSSKADTSRRGDSDSSSMMRSAEEDGEDNNNCLGGETSWAVPQIQSPPTASGLNWPKHFHHHSVFVPDITSSTPYTGSSPNQRVGKRRRRF |
| AT2G21320.1 | AtBBX18 | MRILCDACESAAAIVFCAADEAALCCSCDEKVHKCNKLASRHLRVGLADPSNAPSCDICENAPAFFYCEIDGSSLCLQCDMVVHVGGKRTHRRFLLLRQRIEFPGDKPNHADQLGLRCQKASSGRGQESNGNGDHDHNMIDLNSNPQRVHEPGSHNQEEGIDVNNANNHEHE |
| AT2G24790.1 | AtBBX04 | MASSSRLCDSCKSTAATLFCRADAAFLCGDCDGKIHTANKLASRHERVWLCEVCEQAPAHVTCKADAAALCVTCDRDIHSANPLSRRHERVPITPFYDAVGPAKSASSSVNFVDEDGGDVTASWLLAKEGIEITNLFSDLDYPKIEVTSEENSSGNDGVVPVQNKLFLNEDYFNFDLSASKISQQGFNFINQTVSTRTIDVPLVPESGGVTAEMTNTETPAVQLSPAEREARVLRYREKRKNRKFEKTIRYASRKAYAEMRPRIKGRFAKRTDSRENDGGDVGVYGGFGVVPSF |
| AT2G31380.1 | AtBBX25 | MKIQCDVCEKAPATLICCADEAALCAKCDVEVHAANKLASKHQRLFLDSLSTKFPPCDICLEKAAFIFCVEDRALLCRDCDEATHAPNTRSANHQRFLATGIRVALSSTSCNQEVEKNHFDPSNQQSLSKPPTQQPAAPSPLWATDEFFSYSDLDCSNKEKEQLDLGELDWLAEMGLFGDQPDQEALPVAEVPELSFSHLAHAHSYNRPMKSNVPNKKQRLEYRYDDEEEHFLVPDLG |
| AT2G33500.1 | AtBBX12 | MGTSTTESVVACEFCGERTAVLFCRADTAKLCLPCDQHVHSANLLSRKHVRSQICDNCSKEPVSVRCFTDNLVLCQECDWDVHGSCSSSATHERSAVEGFSGCPSVLELAAVWGIDLKGKKKEDDEDELTKNFGMGLDSWGSGSNIVQELIVPYDVSCKKQSFSFGRSKQVVFEQLELLKRGFVEGEGEIMVPEGINGGGSISQPSPTTSFTSLLMSQSLCGNGMQWNATNHSTGQNTQIWDFNLGQSRNPDEPSPVETKGSTFTFNNVTHLKNDTRTTNMNAFKESYQQEDSVHSTSTKGQETSKSNNIPAAIHSHKSSNDSCGLHCTEHIAITSNRATRLVAVTNADLEQMAQNRDNAMQRYKEKKKTRRYDKTIRYETRKARAETRLRVKGRFVKATDP |
| AT3G02380.1 | AtBBX03 | MLKEESNESGTWARACDTCRSAACTVYCEADSAYLCTTCDARVHAANRVASRHERVRVCQSCESAPAAFLCKADAASLCTACDAEIHSANPLARRHQRVPILPLSANSCSSMAPSETDADNDEDDREVASWLLPNPGKNIGNQNNGFLFGVEYLDLVDYSSSMDNQFEDNQYTHYQRSFGGDGVVPLQVEESTSHLQQSQQNFQLGINYGFSSGAHYNNNSLKDLNHSASVSSMDISVVPESTASDITVQHPRTTKETIDQLSGPPTQVVQQLTPMEREARVLRYREKKKTRKFDKTIRYASRKAYAEIRPRIKGRFAKRIETEAEAEEIFSTSLMSETGYGIVPSF |
| AT3G07650.1 | AtBBX07 | MGYMCDFCGEQRSMVYCRSDAACLCLSCDRSVHSANALSKRHSRTLVCERCNAQPATVRCVEERVSLCQNCDWSGHNNSNNNNSSSSSTSPQQHKRQTISCYSGCPSSSELASIWSFCLDLAGQSICEQELGMMNIDDDGPTDKKTCNEDKKDVLVGSSSIPETSSVPQGKSSSAKDVGMCEDDFYGNLGMDEVDMALENYEELFGTAFNPSEELFGHGGIDSLFHKHQTAPEGGNSVQPAGSNDSFMSSKTEPIICFASKPAHSNISFSGVTGESSAGDFQECGASSSIQLSGEPPWYPPTLQDNNACSHSVTRNNAVMRYKEKKKARKFDKRVRYASRKARADVRRRVKGRFVKAGEAYDYDPLTPTRSY |
| AT3G21150.1 | AtBBX32 | MVSFCELCGAEADLHCAADSAFLCRSCDAKFHASNFLFARHFRRVICPNCKSLTQNFVSGPLLPWPPRTTCCSESSSSSCCSSLDCVSSSELSSTTRDVNRARGRENRVNAKAVAVTVADGIFVNWCGKLGLNRDLTNAVVSYASLALAVETRPRATKRVFLAAAFWFGVKNTTTWQNLKKVEDVTGVSAGMIRAVESKLARAMTQQLRRWRVDSEEGWAENDNV |
| AT3G21880.1 | AtBBX10 | MEPKCDHCATSQALIYCKSDLAKLCLNCDVHVHSANPLSHRHIRSLICEKCFSQPAAIRCLDEKVSYCQGCHWHESNCSELGHRVQSLNPFSGCPSPTDFNRMWSSILEPPVSGLLSPFVGSFPLNDLNNTMFDTAYSMVPHNISYTQNFSDNLSFFSTESKGYPDMVLKLEEGEEDLCEGLNLDDAPLNFDVGDDIIGCSSEVHIEPDHTVPNCLLIDKTNTSSFTGSNFTVDKALEASPPGQQMNINTGLQLPLSPVLFGQIHPSLNITGENNAADYQDCGMSPGFIMSEAPWETNFEVSCPQARNEAKLRYKEKKLKRSFGKQIRYASRKARADTRKRVKGRFVKAGDSYDYDPSSPTTNN |
| AT3G21890.1 | AtBBX31 | MCRGLNNEESRRSDGGGCRSLCTRPSVPVRCELCDGDASVFCEADSAFLCRKCDRWVHGANFLAWRHVRRVLCTSCQKLTRRCLVGDHDFHVVLPSVTTVGETTVENRSEQDNHEVPFVFL |
| AT4G10240.1 | AtBBX23 | MKIQCEVCEKAEAEVLCCSDEAVLCKPCDIKVHEANKLFQRHHRVALQKDAASATTASGAPLCDICQERKGYFFCLEDRAMLCNDCDEAIHTCNSHQRFLLSGVQVSDQSLTENSECSTSFSSETYQIQSKVSLNSQYSSEETEAGNSGEIVHKNPSVILSP |
| AT4G15248.1 | AtBBX30 | MCRGFEKEEERRSDNGGCQRLCTESHKAPVSCELCGENATVYCEADAAFLCRKCDRWVHSANFLARRHLRRVICTTCRKLTRRCLVGDNFNVVLPEIRMIARIEEHSSDHKIPFVFL |
| AT4G15250.1 | AtBBX09 | MEARCDFCGTEKALIYCKSDSAKLCLNCDVNVHSANPLSQRHTRSLLCEKCSLQPTAVHCMNENVSLCQGCQWTASNCTGLGHRLQSLNPYSDCPSPSDFGKIWSSTLEPSVTSLVSPFSDTLLQELDDWNGSSTSVVTQTQNLKDYSSFFPMESNLPKVIEEECSGLDLCEGINLDDAPLNFNASNDIIGCSSLDNTKCYEYEDSFKEENNIGLPSLLLPTLSGNVVPNMSLSMSNLTGESNATDYQDCGISPGFLIGDSPWESNVEVSFNPKLRDEAKKRYKQKKSKRMFGKQIRYASRKARADTRKRVKGRFVKSGETFEYDPSLVM |
| AT4G27310.1 | AtBBX28 | MGKKCDLCNGVARMYCESDQASLCWDCDGKVHGANFLVAKHTRCLLCSACQSLTPWKATGLRLGPTFSVCESCVALKNAGGGRGNRVLSENRGQEEVNSFESEEDRIREDHGDGDDAESYDDDEEEDEDEEYSDDEDEDDDEDGDDEEAENQVVPWSAAAQVPPVMSSSSSDGGSGGSVTKRTRARENSDLLCSDDEIGSSSAQGSNYSRPLKRSAFKSTVVV |
| AT4G38960.1 | AtBBX19 | MRILCDACENAAAIIFCAADEAALCRPCDEKVHMCNKLASRHVRVGLAEPSNAPCCDICENAPAFFYCEIDGSSLCLQCDMVVHVGGKRTHGRFLLLRQRIEFPGDKPKENNTRDNLQNQRVSTNGNGEANGKIDDEMIDLNANPQRVHEPSSNNNGIDVNNENNHEPAGLVPVGPFKRESEK |
| AT4G39070.1 | AtBBX20 | MKIWCAVCDKEEASVFCCADEAALCNGCDRHVHFANKLAGKHLRFSLTSPTFKDAPLCDICGERRALLFCQEDRAILCRECDIPIHQANEHTKKHNRFLLTGVKISASPSAYPRASNSNSAAAFGRAKTRPKSVSSEVPSSASNEVFTSSSSTTTSNCYYGIEENYHHVSDSGSGSGCTGSISEYLMETLPGWRVEDLLEHPSCVSYEDNIITNNNNSESYRVYDGSSQFHHQGFWDHKPFS |
| AT5G15840.1 | AtBBX01 | MLKQESNDIGSGENNRARPCDTCRSNACTVYCHADSAYLCMSCDAQVHSANRVASRHKRVRVCESCERAPAAFLCEADDASLCTACDSEVHSANPLARRHQRVPILPISGNSFSSMTTTHHQSEKTMTDPEKRLVVDQEEGEEGDKDAKEVASWLFPNSDKNNNNQNNGLLFSDEYLNLVDYNSSMDYKFTGEYSQHQQNCSVPQTSYGGDRVVPLKLEESRGHQCHNQQNFQFNIKYGSSGTHYNDNGSINHNAYISSMETGVVPESTACVTTASHPRTPKGTVEQQPDPASQMITVTQLSPMDREARVLRYREKRKTRKFEKTIRYASRKAYAEIRPRVNGRFAKREIEAEEQGFNTMLMYNTGYGIVPSF |
| AT5G15850.1 | AtBBX02 | MLKVESNWAQACDTCRSAACTVYCRADSAYLCSSCDAQVHAANRLASRHERVRVCQSCERAPAAFFCKADAASLCTTCDSEIHSANPLARRHQRVPILPISEYSYSSTATNHSCETTVTDPENRLVLGQEEEDEDEAEAASWLLPNSGKNSGNNNGFSIGDEFLNLVDYSSSDKQFTDQSNQYQLDCNVPQRSYGEDGVVPLQIEVSKGMYQEQQNFQLSINCGSWGALRSSNGSLSHMVNVSSMDLGVVPESTTSDATVSNPRSPKAVTDQPPYPPAQMLSPRDREARVLRYREKKKMRKFEKTIRYASRKAYAEKRPRIKGRFAKKKDVDEEANQAFSTMITFDTGYGIVPSF |
| AT5G24930.1 | AtBBX05 | MDPTWIDSLTRSCEANSNTNHKRKRERETLKHREKKKKRFRERKMASKLCDSCKSATAALYCRPDAAFLCLSCDSKVHAANKLASRHARVWMCEVCEQAPAHVTCKADAAALCVTCDRDIHSANPLARRHERVPVTPFYDSVSSDGSVKHTAVNFLDDCYFSDIDGNGSREEEEEEAASWLLLPNPKTTTTATAGIVAVTSAEEVPGDSPEMNTGQQYLFSDPDPYLDLDYGNVDPKVESLEQNSSGTDGVVPVENRTVRIPTVNENCFEMDFTGGSKGFTYGGGYNCISHSVSSSSMEVGVVPDGGSVADVSYPYGGPATSGADPGTQRAVPLTSAEREARVMRYREKRKNRKFEKTIRYASRKAYAEMRPRIKGRFAKRTDTNESNDVVGHGGIFSGFGLVPTF |
| AT5G48250.1 | AtBBX08 | MGYMCDFCGEQRSMVYCRSDAACLCLSCDRNVHSANALSKRHSRTLVCERCNAQPASVRCSDERVSLCQNCDWSGHDGKNSTTTSHHKRQTINCYSGCPSSAELSSIWSFCMDLNISSAEESACEQGMGLMTIDEDGTGEKSGVQKINVEQPETSSAAQGMDHSSVPENSSMAKELGVCEDDFNGNLISDEVDLALENYEELFGSAFNSSRYLFEHGGIGSLFEKDEAHEGSMQQPALSNNASADSFMTCRTEPIICYSSKPAHSNISFSGITGESNAGDFQDCGASSMKQLSREPQPWCHPTAQDIIASSHATTRNNAVMRYKEKKKARKFDKRVRYVSRKERADVRRRVKGRFVKSGEAYDYDPMSPTRSY |
| AT5G54470.1 | AtBBX29 | MGKKKCELCCGVARMYCESDQASLCWDCDGKVHGANFLVAKHMRCLLCSACQSHTPWKASGLNLGPTVSICESCLARKKNNNSSLAGRDQNLNQEEEIIGCNDGAESYDEESDEDEEEEEVENQVVPAAVEQELPVVSSSSSVSSGEGDQVVKRTRLDLDLNLSDEENQSRPLKRLSRDEGLSRSTVVMNSSIVKLHGGRRKAEGCDTSSSSSFY |
| AT5G57660.1 | AtBBX06 | MGFGLESIKSISGGWGAAARSCDACKSVTAAVFCRVDSAFLCIACDTRIHSFTRHERVWVCEVCEQAPAAVTCKADAAALCVSCDADIHSANPLASRHERVPVETFFDSAETAVAKISASSTFGILGSSTTVDLTAVPVMADDLGLCPWLLPNDFNEPAKIEIGTENMKGSSDFMFSDFDRLIDFEFPNSFNHHQNNAGGDSLVPVQTKTEPLPLTNNDHCFDIDFCRSKLSAFTYPSQSVSHSVSTSSIEYGVVPDGNTNNSVNRSTITSSTTGGDHQASSMDREARVLRYREKRKNRKFEKTIRYASRKAYAESRPRIKGRFAKRTETENDDIFLSHVYASAAHAQYGVVPTF |
| AT2G47890.1 | AtBBX11 | MEAEEGHQRDRLCDYCDSSVALVYCKADSAKLCLACDKQVHVANQLFAKHFRSLLCDSCNESPSSLFCETERSVLCQNCDWQHHTASSSLHSRRPFEGFTGCPSVPELLAIVGLDDLTLDSGLLWESPEIVSLNDLIVSGGSGTHNFRATDVPPLPKNRHATCGKYKDEMIRQLRGLSRSEPGCLKFETPDAEIDAGFQFLAPDLFSTCELESGLKWFDQQDHEDFPYCSLLKNLSESDEKPENVDRESSVMVPVSGCLNRCEEETVMVPVITSTRSMTHEINSLERNSALSRYKEKKKSRRYEKHIRYESRKVRAESRTRIRGRFAKAADP |
| AT5G68190.1 | AtBBX27 | MLCIIIIENMERVCEFCKAYRAVVYCIADTANLCLTCDAKVHSANSLSGRHLRTVLCDSCKNQPCVVRCFDHKMFLCHGCNDKFHGGGSSEHRRRDLRCYTGCPPAKDFAVMWGFRVMDDDDDVSLEQSFRMVKPKVQREGGFILEQILELEKVQLREENGSSSLTERGDPSPLELPKKPEEQLIDLPQTGKELVVDFSHLSSSSTLGDSFWECKSPYNKNNQLWHQNIQDIGVCEDTICSDDDFQIPDIDLTFRNFEEQFGADPEPIADSNNVFFVSSLDKSHEMKTFSSSFNNPIFAPKPASSTISFSSSETDNPYSHSEEVISFCPSLSNNTRQKVITRLKEKKRARVEEKKA |
| LOC_Os01g10580.1 | OsBBX01 | MKVLCSACEAAEARVLCCADDAALCARCDLHVHAANRLAGKHHRLPLLSSSSSSSSPSPPTCDICQDAHAYFFCVEDRALLCRACDVAVHTANALVSAHRRFLLTGVHVGLDAAADDDDKHPPHPLSSSLPRNTAPPPQPPPKRSPSPIYSDDDVIDWATGGHDIGITGNLPDWSLVDEQFNTPALPPVVTKTPPKRASRGPVTAGTAAAVFGNLAGGSPDWPLNEFFGFADFSSGFGFAENGTSKADSGKIGSMDGSPNGGRSSSSSSSSSAAAAGGGGGGQDFFGQVPEVHWAVPELPSPPTASGLHWQRDPRYGGGATDASAVFVPDISSPENPFRCFAAAAAGDHTMKRRRRC |
| LOC_Os02g07930.1 | OsBBX02 | MEVGNGKCGGGGAGCELCGGVAAVHCAADSAFLCLVCDDKVHGANFLASRHRRRRLGVEVVDEEDDARSTASSSCVSTADSASSTAAAAAAVESEDVRRRGRRGRRAPRAEAVLEGWAKRMGLSSGAARRRAAAAGAALRAVGRGVAASRVPIRVAMAAALWSEVASSSSRRRRRPGAGQAALLRRLEASAHVPARLLLTVASWMARASTPPAAEEGWAECS |
| LOC_Os02g08150.1 | OsBBX03 | MEMELGLGRYWGVGRRRCGACAVAPAAVHCRTCDGDGGGGGYLCAGCDAEHGRAGHERVWVCEVCELAPAAVTCKADAAALCAACDSDIHDANPLARRHERVPVHPIGSSAAPPPDALLLGGENDAAAAVDGGGGGKEVKLDFLFADFMDPYLGGSPELARFPHADSVVPNHNGSAGPAMELGFAGGGGAAVKPSYSSYTAASLGNSGSSSEVGLVPDAICGGGGGGIIELDFAQSKAAYLPYASTPSHSMSSSMDMGVAAPEMSDCAAAAAGRAYAAEGRAARLMRYREKRKNRRFEKTIRYASRKAYAETRPRVKGRFAKRADDHDAAAPPPQIMLDFAGYGVVPTF |
| LOC_Os02g39360.1 | OsBBX04 | MKIQCDACESAAAAVVCCADEAALCAACDVEVHAANKLAGKHQRLPLEALSARLPRCDVCQEKAAFIFCVEDRALFCRDCDEPIHVPGTLSGNHQRYLATGIRVGFASASPCDGGSDAHDSDHHAPPMGSSEHHHHHQQPAPTVAVDTPSPQFLPQGWAVDELLQFSDYETGDKLQKESSPPLGFQELEWFADIDLFHNQAPKGGAAAGRTTAEVPELFASQAANDVAYYRPPTRTAAAAFTAATGFRQSKKARVELPDDEEDYLIVPDLG |
| LOC_Os02g39710.1 | OsBBX05 | MEAVEDKAMVGVGGAVAAGYSSSSWGLGTRACDSCGGEAARLYCRADGAFLCARCDARAHGAGSRHARVWLCEVCEHAPAAVTCRADAAALCAACDADIHSANPLARRHERLPVAPFFGPLADAPQPFPFSQAAADAAAAREEDADDDRSNEAEAASWLLPEPDDNSHEDSAAAADAFFADTGAYLGVDLDFARSMDGIKAIGVPVAPPELDLTAGSLFYPEHSMAHSLSSSEVAIVPDALSAGSAAPPMVVVVASKGKEREARLMRYREKRKNRRFDKTIRYASRKAYAETRPRIKGRFAKRTADADDDDEAPCSPAFSALAASDGVVPSF |
| LOC_Os02g43170.1 | OsBBX06 | MKVQCDVCAAEAASVFCCADEAALCDACDHRVHRANKLAGKHRRFSLLNPSASGRSPTSTTAPLCDICQEKRGFLFCKEDRAILCRECDVPVHTASELTMRHSRYLLTGVRLSSEPAASPAPPSEEENSSSFCCSADDAVPAPAAPATSHGGSSGSSSISEYLTTLPGWHVEDFLVDDATAEAAAAAAATSSGISANGPCQGVTRIGGLQESAGYPAWMAQQQLCCDGLVAGDASPASRERWVPQMYADQLAAGSKRSRTSTASSYSYW |
| LOC_Os02g49230.1 | OsBBX07 | MDALCDFCREQRSMVYCRSDAASLCLSCDRNVHSANALSRRHTRTLLCDRCVGQPAAVRCLEENTSLCQNCDWNGHGAASSAAGHKRQTINCYSGCPSSAELSRIWSFSMDIPTVAAEPNCEEGINMMSINDNDVNNHCGAPEDGRLLDIASTALMSDLPTGDKFKPLIGSSSGDGMNLLPLNSDQPAEPVSTTPKAPCVTDKDMFNDGSVYGDFCVDDADLTFENYEELFGTSHVQTEQLFDDAGIDSYFEMKDVPADESNEQPKPVQPECSNVASVDSGMSNPAARADSSHCIPGRQAISNISLSFSGLTGESSAGYFQDCGVSSMILMGEPPWHPPGPESSSAGGSRDNALTRYKEKKKRRKFDKKIRYASRKARADVRKRVKGRFVKAGEAYDYDPLSQTRSY |
| LOC_Os02g49880.1 | OsBBX08 | MSCSSEKAAGAVGGKAARACDSCLRRRARWYCAADDAFLCQGCDTSVHSANPLARRHERLRLRVSSPPPLTARASVEEEAAAAVGTTTTTTSKREGGVTPAWSKRKARTRRPQVKSVGQLLSRRLVVPEMAVESSDERKADEDGAHEELEGQLLYRVPVFDPSLAEFCSPPPIDDAAAASSSCFKEDAADGAVEDAKYPAAAASSPVQQLPDSFVNFEPTDAELREFAADMEALLGQGLDDSNELQDSFYMETLGLITPPVEESGRVKMELDGGVASNSRVSLPSCRAHPKPEDVESADVLDIDFNCTSPDEQKSSASNGAAADSQFFHRSLDLRLNYEAIIESWGNSPWTDGRPPHGQLDDFWPNDHHYSGLWAAGGGGHGAEVGMMTVRPRMDGPGREARVTRYREKRRTRLFSKKIRYEVRKLNAEKRPRMKGRFVKRPSAAAAPCAVT |
| LOC_Os03g22770.1 | OsBBX09 | MGQDEVEVGAEKKDQELPEVEVVEEEEEEGSKKAAAGCDYCGDAAAVVYCRADAARLCLPCDRHVHGANGVCSRHARAPLCAACAAAGAVFRRGAGGFLCSNCDFSRHRHGGERDPAAPLHDRSTVHPYTGCPSALDLAALLGISYSDKAAAATAAAGGDDGGWWAIWEEPQVLSLEDLIVPTTSCHGFEPLLTPSSPKIQNSPDGKVNEEVIRQLTELANSDGGGAQIWAHREAAQAGDHQLPSWGTTTQHNTGHGNFGTANSNEVATMPTPGYENGGWDNSDYPALNDPCKVEFTYEQPPASSAEACISSFVQMSELCPSMSNGSSMEETHQTNPGNGTPMQVLPKMPEFVPCPDRNLVISRYKEKRKTRRFDRQVRYESRKARADSRLRIKGRFAKVNQI |
| LOC_Os03g50310.1 | OsBBX10 | MASAAAATGAALGARTARACDGCMRRRARWHCPADDAFLCQACDASVHSANPLARRHHRVRLPSASSSPASSPRSAAAPRAGSDDPDAPAWLHGLKRRPRTPRTKPGGGGKHDASAATVAAAAASAVPDLEAEESGIVGDTDHDVGEEDDEDLLYRVPVFDPMLAELYNPVAADDEEQQIEQKPAARVVPFSEPSPEFASGSVEADGLSGFDVPDMELASFAADMESLLMGVDEGFDDLGFLDDEKPHVKLDLDMDMDFASISPAPAPEREERKRKRPEMILKLDYEGVIDSWARDGASPWFHGERPRFDPSESWPDFPAGSRGGLGAAVTAVTGGEREARVSRYREKRRTRLFAKKIRYEVRKLNAEKRPRMKGRFVKRAAALPPLPLPRHQHPPPPPPRALPPVPMMLAPRGAHGRYRF |
| LOC_Os04g41560.2 | OsBBX11 | MRIQCDACEAAAATVVCCADEAALCARCDVEIHAANKLASKHQRLPLDAALPAALPRCDVCQEKAAFIFCVEDRALFCRDCDEPIHVPGTLSGNHQRYLTTGIRVGFSSVCSANADHLPPPAPKGNSKPPASGIAAAAAPKPAVSAAAQEVPSSPFLPPSGWAVEDLLQLSDYESSDKKGSPIGFKDLEWLDDIDLFHVQSPAKGGSTAAEVPELFASPQPASNMGLYKASGARQSKKPRVEIPDDDEDFFIVPDLG |
| LOC_Os04g42020.1 | OsBBX12 | MEGDDKSAVVGGAYWGLAARACDACGGEAARLFCRADAAFLCAGCDARAHGPGSRHARVWLCEVCEHAPAAVTCRADAAALCAACDADIHSANPLARRHERLPVAPFFGALADAPKPGSGAHGGDAAAADDDGSNDAEAASWLLPEPDHGQKDGAVGATDELYADSDPYLDLDFARSMDDIKAIGVQNGPPELDITGGKLFYSDHSMNHSVSSSEAAVVPDAAAGGGAPMPVVSRGREREARLMRYREKRKSRRFEKTIRYASRKAYAETRPRIKGRFAKRTKGGAGADADADADADGEDEEMYSSAAAAVAALMAPGGSDADYGVDGVVPTF |
| LOC_Os04g45690.1 | OsBBX13 | MKVQCDVCAAEAASVFCCADEAALCDACDRRVHSANKLAGKHRRFSLLQPLASSSSAQKPPLCDICQEKRGFLFCKEDRAILCRECDVTVHTTSELTRRHGRFLLTGVRLSSAPMDSPAPSEEEEEEAGEDYSCSPSSVAGTAAGSASDGSSISEYLTKTLPGWHVEDFLVDEATAASSSSDGLFQGGLLAQIGGVPDGYAAWAGREQLHSGVAVAADERASRERWVPQMNAEWGAGSKRPRASPPCLYW |
| LOC_Os05g11510.1 | OsBBX14 | MSPPPPPYYHHLLLLRSSPTTTGGGARVLAAAELARMKLLCSACEAAEASVLCCADEAALCARCDRDIHAANRLAGKHLRLPLLSPASSSSSSAAALAPPPPSPPKCDICQESHAYFFCLEDRALLCRSCDVAVHTANAFVSAHRRFLLTGVQVGQEQDEHSPDPPEPSPPPPPPPPASKSDHPAPLYGEGGGGFSWDAADSPAAGGLPDWSAVVDQFGSPPPPRHTDTATVTTPPPTKRSPRAPAFGGQGGMMDWPLGEFFGGFTDFTGGFGFGFGDSGTSKADSGKLGGSTDGSPYYRSSSEDDRNADELFGQVPEIQWSVPELPSPPTASGLHWQRHPAATHGGGGGGPDTTAFVPDICSPDSCFPATTSKRRRQ |
| LOC_Os06g01340.1 | OsBBX15 | MENEVGCECQLCGGRRGVVFCGAHGGRLCLQCDRALHQAHGGAGDHPRAPLCDSCNAAAAELRLNDGATLCGPCAYPYAYAYPYTYTYVYTGCPTPLEMMRLLHAAPPPPPATCSLQQRGEGEELLPTLLSATATPNTATAAPMAMPPPPLQHHTTTSLIMMIRNIHKREERNRAKLRFSKQIKYACRKAGADARKRVKGRFAKASSSSSSSSSSSSSIDHRL |
| LOC_Os06g05890.1 | OsBBX16 | MKIQCNACGAAEARVLCCADEAALCTACDEEVHAANKLAGKHQRVPLLSDDGGAAPAAAAPAVPKCDICQEASGYFFCLEDRALLCRDCDVSIHTVNSFVSVHQRFLLTGVQVGLDPADPVPPVADKHVKSAGGSVDSATKHLQRNPTDLSGENSASLPSQNVINGNYSRQSSVTMAKTGQVNWTMSNNTIRSIDPPPKYSSEESPALLLASHTSTMAAYSSQISKDSDRIYNLPFTGGNGSDSLHDWHVDEFFSNSEFGFAEHGSSKGDNAKPGSAGGSPQCRLAEGLFVEGLLGQVPDNPWTVPEVPSPPTASGLYWQNNLLCPSYDSTMFVPEISSLENSQNNFTVSAGLKRRRRQF |
| LOC_Os06g15330.1 | OsBBX17 | MSSTAKAAAAGAVGAKSARACDGCLRRRARWYCAADDAFLCQGCDTSVHSANPLARRHERLRLRPSSPPPLVPPSGSGRRDEAVPAAWFKRKARTPRSHAAKSAAAFGQLLSRRLVVVPEAAAGSGGDSPEERKDEGEIVEEQEQLLYRVPIFDPALSEFCSPPPLEDAAAAVSCCNEDGAVENPTKPSMTTTTATTPPLQFFPDGQANFGPTDAELREFAADMEALLGRGLDDGNDEDSFCMETLGLIEPVDDDAGRVKVEADGDAGMTLAWCHELDTETSSGEMLDIDFDCGSPQAATTPDEKVGSSGPAAADDDAQLQQSNLALSLNYEAIIESWGTSPWTDGERPHVKLDDSWPRDYSGVWMAAAGVFGHGGEEQALTPRLGMDGGREARVSRYREKRRTRLFSKKIRYEVRKLNAEKRPRMKGRFVKRAAAAATAAVATACVA |
| LOC_Os06g16370.1 | OsBBX18 | MNYNFGGNVFDQEVGVGGEGGGGGEGSGCPWARPCDGCRAAPSVVYCRADAAYLCASCDARVHAANRVASRHERVRVCEACERAPAALACRADAAALCVACDVQVHSANPLPAITIPATSVLAEAVVATATVLGDKDEEVDSWLLLSKDSDNNNNNNNNNDNDNNDNNNSNSSNNGMYFGEVDEYFDLVGYNSYYDNRIENNQDRQYGMHEQQEQQQQQQEMQKEFAEKEGSECVVPSQITMLSEQQHSGYGVVGADQAASMTAGVSAYTDSISNSISFSSMEAGIVPDSTVIDMPNSRILTPAGAINLFSGPSLQMSLHFSSMDREARVLRYREKKKARKFEKTIRYETRKAYAEARPRIKGRFAKRSDVQIEVDQMFSTAALSDGSYGTVPWF |
| LOC_Os06g19444.1 | OsBBX19 | MGALCDFCGEQRSMVYCRSDAASLCLSCDRNVHSANALSRRHTRTLLCDRCASQPAMVRCLVENASLCQNCDWNGHSAGSSAAGHKRQTINCYSGCPSSSELSKIWTFVSDIPNVAPEPNCEQGISMMSISDSGVSNQDNAAGDSSLLDIASATLMSDLGTAGKPKSLIGSSSEAGVNLLPLATDQMAGSVDSTSAKVPYTADQDMFSKDSIYEDFCVDDVDLSFENYEELFGTSHIQTEQLFDDAGIDSYFESKEIPSGNSDEQPKLMQPVTSNAVSADSGMSIPGAKGDSSLCIPVRQARSSISLSFSGLTGESSAGDYQDCGVSPVLLMGEPPWHPPGPEGSFAGATRDDAITRYKEKKKRRKFDKKIRYASRKARADVRKRVKGRFVKAGEAYDYDPLCETRSY |
| LOC_Os06g44450.1 | OsBBX20 | MMELRKYWGVGGRRCGACEASPAAVHCRGCGGVYLCTACDARPGHARAAHERVWVCEVCEVAPAAVTCKADAAVLCAACDADIHDANPLARRHARVPVAPIGSAAAAAVAAEAMLFGVAAAGAEAEAVEDKAAAEHHHHQQRQQHGALNLNVEAKDMKLDYLFSDLDPYLNVEFARFPHADSVVPNGAGAGAAIELDFTCGLGVGVGGAKQSYSSYTATDLAHSGSSSEVGVVPEAMCGGGGAIDLDFTRPKPQPYMPYTATPPPSHSVVSAQMSSSVVDVGVVPERAAAMGEGREARLMRYREKRKNRRFEKTIRYASRKAYAETRPRIKGRFAKRADHDADDADADADDPAAVPSSYMLDFGYGVVPSF |
| LOC_Os06g45040.1 | OsBBX21 | MGGEAERCALCGAAAAVHCEADAAFLCAACDAKVHGANFLASRHHRRRVAAGAVVVVEVEEEEGYESGASAASSTSCVSTADSDVAASAAARRGRRRRPRAAARPRAEVVLEGWGKRMGLAAGAARRRAAAAGRALRACGGDVAAARVPLRVAMAAALWWEVAAHRVSGVSGAGHADALRRLEACAHVPARLLTAVASSMARARARRRAAADNEEGWDECSCSEAPNALGGPHSFRTTEAPAFSFIASVRWKKRLLIVLAEYKYATSNQFPTLN |
| LOC_Os06g49880.1 | OsBBX22 | MRVQCDVCAAEPAAVLCCADEAALCSACDRRVHRANRLASKHRRLPLVHPSSSSSGDGGAAAAPLCDVCREKRGLVFCVEDRAILCADCDEPIHSANDLTAKHTRFLLVGAKLSPAALAEQPLPSSDCSSDDDAAAAATEEEYHSSAASTGAAVSAPLDASSNGAGGGGGVGGSSISDYLTTICPGWRVEDLLPDDDAFAAAAAQAGKEKDERVPFLDADLFDVVAGRPEKKGGAWAPHVPHLPAWCLDEVPVVVAASAAPAATPVKAKQGHVRDSHWSDSDAFAVPEFSPPPPPAKRARPSSQFWCF |
| LOC_Os07g47140.1 | OsBBX23 | MARDDDPAKKLAVDGGVAAAARCCDFCGGLPAVVYCRADSARLCLPCDRHVHAANTVSTR  HARAPLCSACRAAPAAAFHRGDGFLCSSCDFDERLRRGSIGGGGDELPLDDRAAVEGYTGCPSIGELAAILGVVGGDSDKPADDGWWSASWEEEAPQVLSLDDIIVPTTSCHGLRPLLTPPSPENQSSPDNGELDGEVVRQLGELARSEAAAQATFVAGDQLASWASPEFTSGHGDFGIEAASTTVPSCENETWIMSTDCTDPTDASKTDIAREEAPASSSAEPCLSSLVEISEICRSMSYSGSGIDNGGHDPSTLAIMPTQALPKKGVYDIAYPDRGTVISRYKEKRKNRRFDKQIRYESRKARADGRLRIKGRFAKSN |
| LOC_Os08g08120.1 | OsBBX24 | MSVAAEGKEKGVGGGGGGAGAGACELCGAAARVYCGADEATLCWGCDAQVHGANFLVARHARALLCRGCARPTPWRAAGPRLGPTASLCERCVRRGGGGRGGGGGGGAAGGGGRGGGGDEEMGGEGDEEEEDEDEEVVVEEEEDEDDEDEEGEGEGENQVVPWAEEAEATPPPVASSTSSSSREAAANGANAADRVKEDQPCSTSQPSLCRYASSAHHGGGGRSDEATSSRNGGGVGGRFLASRHRKRSPSDFRRSGLAQSVSGVQGRNCSNAVVGRNDASMM |
| LOC_Os08g15050.1 | OsBBX25 | MMASDGSASPASCGGAACGVCGGAATVYCAADAAALCVPCDAAVHAANPLASRHDRVPLAVAMAAASSGVYDHLFAPDDDAASSWAAAAAAGAAVQGQGQGSPNDSSSSFTNDSAGGGGGGGAERSLFDLLSDVDIMSCGGGGLASSFDGAAAPPLWLHPGQLAALTPWSPADSVVVPTSAAGAVAAAAAAREERVRRYREKRKNRKFQKTIRYASRKAYAEARPRIKGRFVKRATTAAASSSSDDDSTAAAGVSGAGGAGAAATKEAKFWLSFSDDGRADGVGFYMDSTTAATAAYGVVPTF |
| LOC_Os08g42440.1 | OsBBX26 | MKDGGGGGGRGQQQQWPCDYCGEAAAALHCRADAARLCVACDRHVHAANALSRKHVRAPLCAACAARPAAARVASASAPAFLCADCDTGCGGDDGAALRVPVEGFSGCPAAAELAASWGLDLPGGCGGEEEEADDAFFSALDYSMLAVDPVLRDLYVPCDPPEVVVAGGGRRLKGEALGHQLAEMARREAETAHPHTQPHSDLSPRTPRRTSAAASGRLQEKQAPPPLPHAAATAAPLPYTSLLMMAPANCTELMENNRVGDEDENVLWESTAPSVPPTQIWDFNLGKSRDHNENSALEVGFGSNNGGFMIKSYNDMLKEISSGTTKDLEDIYDSRYFAAAEDIMSTNVCQLSSKNPSTRSNKRKASSCASTIDGPTTSTSHVPAASGALGGSSNDRGSALPKEISFCDQTVVPTGADQRPCTIKIDSETLAQNRDSAMQRYREKKKNRRYEKHIRYESRKLRADTRKRVKGRFVKSNGAPDDVSNGG |
| LOC_Os09g06464.1 | OsBBX27 | MLKLEPEFPGLPQRCDSCRSAPCAFYCLADSAALCATCDADVHSVNPLARRHRRVPMGVVAAPGAGGAFVVRPAGGVNSSWPIREGRRCDYDDDDADAAGEEDEEATSWLLFDPLKDSSDQGLPPFGDALVADFLNLGGGAGEKEDASSSKDCSSSHGKSSEGSHEFAVPGEPVPERQGFGAVSMDITDYDASNFRRGYSFGASLGHSVSMSSLENMSTVPDCGVPDITTSYLRSSKSTIDLFTAAAGSPVAAHSIMSPPQFMGAIDREARVHRYREKRKTRRFEKTIRYASRKAYAETRPRIKGRFAKRSDTDLEVDQYFSTTADSSCGVVPTF |
| LOC_Os09g33550.1 | OsBBX28 | MTWRSCDYCGEAAAALHCRADAARLCVACDRHVHGANALSRRHVRAPLCARCEARPAAARVAAVAGAGGCGGGGEARFLCAGCADDDGAEAARVPVVGFSGCPGAAELAASWGLDLGGGGGRDEFEEDPFFPEAGYPMLAADRVLRDMYVPCDPPPEVAAGGRGRRLKGDSLCHQLAELARREMESAPAQANSGSISPSARRGSAAAIRHEAAAAAAAQRATLPYKSTPVTEAAGCGDVG  NGEQFTDDNELVWQRTAPSDPPCQIWDFNLGKSRDHDEHSALELHFGPKDGGFMIKSYNDMIEEVSSSSRKDLQYIYDSTYSFATEDIVSANIYQLTPKQLSTATSGNRRHKNEQHGLTNDGPSSSRIVDVDRTLNSSPEEVAAVLAGENCITDQTVTGADQRNSLKIDSKTIAMNRDNAMQRYREKRKTRRYDKHIRYESRKMRADTRTRVKGRFVRATDIFNVGGGDGG |
| LOC_Os09g35880.1 | OsBBX29 | MRTICDVCESAPAVLFCVADEAALCRSCDEKVHMCNKLARRHVRVGLADPNKVQRCDICENAPAFFYCEIDGTSLCLSCDMTVHVGGKRTHGRYLLLRQRVEFPGDKPGHMDDVAMQQKDPENRTDQKKAPHSVTKEQMANHHNVSDDPASDGNCDDQGNIDSKMIDLNMRPVRTHGQGSNSQTQGVDVSVNNHDSPGVVPTCNFEREANK |
| LOC_Os12g10660.1 | OsBBX30 | MKIGCDACEQAEAAVLCCADEAALCRRCDAAVHSANRLAGKHTRVALLLPSSSSAAAGDDDHHPTCDICQEKTGYFFCLEDRALLCRSCDVAVHTATAHAAAHRRFLITGVRIGGSVDAAAAADVIVSPTSSSIAPAGSASSNHAGAAGNNNGRSPAPVRFSGGDGGVEPEQQWPWSDVFAADDDDDVSAAMEQCYYHGISEPHSSSLTG |
| PH02Gene16459.t1 | PeBBX04 | MCHSLDSTATLLLVLHTELPTLTTPTHSQEAGGTSFLWARPCDGCHAAPSAVYCRADAAYLCASCDTQVHSANHVASRHERVRVCEACESAPAVLACRADAAELCTTCDAQVHSANPIAQRHQRVPVLPLPAAAIPAASGFAEAEAAVTAHGNKEEEEEANSWLLLSRDSDDNNCTNNNMYFGEVDQYFDLVGYNLYHDNSVTSNPEEQYKMQEQQHVQKRYREKGGSECVVPSQVAMASEQQQSGYGIIGAEQAASMAAGVSAYTASISNSVS |
| PH02Gene27822.t1 | PeBBX02 | MDAEDSKPVVGAGYWGLGARPCDSCGVDAARLYCSADGAFLCTGCDARAHGAGSRHARVWLCEVCEHAPAAVTCRADAAALCAACDADIHSANPLARRHERLPVAPFFGALADAPQPFPSPAFAAAAATVTGPQVDADDDGSNEAEAASWLLPEPDNSHEGSAAADAFFAESDAYLGLDLDFARSMDGIKAVGVPAVPPELDLAAGNLFYTEHSMNHSVSSSEVAVVPDALSAGAAPAPSLAVVASKGKEREARLMRYREKRKNRRFNKTIRYASRKAYAEMRPRIKGRFAKRSAEDDALLEHDGACSPAVSALVASDGDYGVVPSF |
| PH02Gene35355.t1 | PeBBX01 | MRTICDVCESAAAVLFCAADEAALCRPCDEKVHMCNKLANRHVRVGLADPNKVPRCDICENSPAFFYCEIDGTSLCLSCDMAVHVGGKRTHGRYLILRQRVEFPGDKPGHMDDVAMQQKDPENQRDQKKAPHSVTKEQMANHHIVSDDLASDGNCDDQGNIDSKMIDLNMRPVRTHGQGSNSQTQGVDLSVNNHESPGAVPTCNFERYSNK |
| PH02Gene39681.t1 | PeBBX03 | MRVQCDVCAAEAASVFCCADEAALCDGCDRRVHRANKLSGKHRRFSLLYPSPSSSASTQPPRPLCDICQEKRGFLFCKEDRAILCRECDVPVHMASKLTMRHSRFLLTGVRLSSEPAASPAPPSEEENSSSFCCSGDDVAPPVPATSHGGSSSISEYLIKTLPGWHVEDFLVDDASAAAAAAAATSTGISSDGSYQGVARIGGLQEAGYPAWMAQEQLFCDSAVAADALASRERWVPQMMYTELAGSKRSRTSTSYSYW |
| PH02Gene45949.t1 | PeBBX05 | MSSSDRKAAGAVGGKAARPCDSCLRRRARWYCAADDAFLCQGCDTSVHSANPLARRHERLRLRASSPPPRSVEGVAAATTTSKRQGVAPAWSKRKARTRRPQVKSVGQLLSRRLVVPEVAVESSEERKAEEDGAEEEQLLHRVPTFDPALAEFCSPLLIDDAAATTSCCREEVDGAVEDTREPVVVASPVQQLPDSFANFGPTDAELREFAADMEALLGRGLDDSNELDDPFNMETLGLVSPAEDGGRVKVEPDGVVSNSGGAVASGPELKSEASAEVLDIDFNCSSPMVVDDDDGFEQKTSASNGGAADAQFFQRSLDLRLNYEAIIESWERSPWTDGERPHVKFDNFWFHDHSGMWMAGGARHGEEAGTLRLRMDGAGREARVTRYREKRRTRLFAKKIRYEVRKLNAEKRPRMKGRFVKRSAGGGAAVAAAPCVVT |
| PH02Gene05295.t1 | PeBBX07 | MSSSAKLAAGAMGGKAARACDGCLRRRARWYCAADDAFLCQGCDSSVHSANPLARRHERLRLRQLSPLLPPSAGAASRARRGGDEVVPAWFKRKARTPRSHAKSVEQLLSRPLVVPEGAVGDSPEERKCEGEIEEEELLYRVPIFDPTLAEFCSPPPLEDVAAAGSCYNENGAVENPTKPAVTPPQMQFFPDSFGPTDAELREFAADMEALLGRGLDDGNEEDSFYMETLGLIEPTEADGGRVKQETDVKARGMLACGLELEPEASGEMLDINFNYGSPQATPDEKAASTDGSTDAQFLQRSLALSLNYEAIIASWGSSPWTDGERPHVKLEDCWPHDYSGVWMVGGGVVGHGGDGVGMPRLGMDGGREARVTRYREKRRTRLFSKKIRYEVRKLNAEKRPRMKGRFVKRATAGGSVA |
| PH02Gene19468.t1 | PeBBX06 | MKIQCNACGTAEARVLCCADEAALCTACDEEVHAANKLAGKHQRVPLLSDGASAAPAAAPDVPRCDICQEASGYFFCLEDRALLCRDCDVAIHTVNSFVSVHQRFLLTGVQVGLDPADPVPPIADKHANAAGGSMDSPTKHLPRRNPTVLFSGESTAPSQNAINGDYSRQNSVPNTRKGVVDWTMNNSAIRSVEPPPKYLSEETPTLLLSSQTTTAFSNQINRDSDRGYNLPFSGGDSLPDWPVDEFFGNSEYGPNFGFAEHGSSKGDNAKLGSAGGSPQCRLAEGLVAEELLDMPGLDADESLNRVPENPWAVPEVPSPPTASGLYWQGNLRYPVYDSTMFVPEISSLDISQNHFTVSAGLKRRRRQF |
| PH02Gene19765.t2 | PeBBX08 | MRVQCDVCGQEPAAVLCCADEAALCSACDRRVHRANRLAGKHRRLPLLHPSLPTNDSDTAAPPLCDVCKDRRGFVFCVEDRAILCADCDEPIHSANELTAKHSRFLLVGAKLSAALVDQAIPSPDGSSDEPHDLAACAAEDSYLDASHGGGGGGGGSSISDYLTNICPGWRVEDLLLDDAAAAAAAAAKGRDEQVPFLDADLSDVVAGRPEKRGAWAPHVPQTPAPPWGLEKVPAAVAAPAAAKAKQGHVRERHWSDSDSDAFAVPEISPSPAKRARPSSFWCF |
| PH02Gene02845.t1 | PeBBX11 | MRVQCDVCGQEPAAVLCCADEAALCSACDRRIHQANKLAGKHRRLPLLHPSSPTNDTAAPPLCDVCKERRGFVFCVEDRAILCAGCDEPIHSANELTAKHSRFLLVGAKLSAPFADQEIPSPEGGSDVAAEDSSLVDSHGGGGGGSSSISDYLTNICPGWRVEDLLLDDAAFAAAAAAKGRDEQVPFLDADLFDVVAGRPEKRGAWAPHVPQTPTLAWGLEEVLAAMAAPATAKTKQGHVRERYWSDSDALAVPEISPPPAKRARPSSFWCF |
| PH02Gene21910.t1 | PeBBX10 | MNYNFGGTVYEQEVAGGEGSCPWARPCDGCNAAPSVVYCRADAAYLCASCDSRVHAANRVATRHERVRVCEACERAPAVLACRADAAVLCVSCDAQVHSANPLARRHQRVPVVPLPAAAIPAASVLAEAAAAATTVLGDKEEEVDSWLLLSKDSDNQNCSSNNNNNSMYFGEVDEYFDLVGYNSYYDNRIDNNQEQYGMQEQQQQQQQEMQKEFAEKEGSECVVPSQVAMVSEQQQQSGYVGAEQAASMTAGVSAYTDSISNSISFSSMEVGIVPDNTVIDMPNSSILTPAGAINLFSGPSLQMPLHLSTMDREARVLRYKEKKKTRKFEKTIRYATRKAYAEARPRIKGRFAKRSDVEIEVDQMFSSAALSDCSYSTVPWF |
| PH02Gene25549.t1 | PeBBX09 | MKIQCNACGAAEARVLCCADEAALCNACDEEVHAANKLAGKHQRVPLLSDGAPAAPAVPKCDICQIAMGN |
| PH02Gene42827.t1 | PeBBX12 | MKVLCSACEAAEARVICCADEAALCARCDRDVHAANRFAGKHQRLPLLAPGAAAPVSSPPKCDICQECHAYFFCLEDRALLCRSCDVAVHTANAFVSAHRRFLLTGVQVGQEEDDHSPEPSPPPPTKSDPAPLYGEGDISWAAATPDATGVTESLPDWSIVNEQFGSPAPRHAEAASRTPPKQSPRAPEFGGQGGMMDWPLGEFFGGFSDFNGGFGFGESGTSKADSGKLGGSTDGSPYYRSSLENRNADEIFRQVPEIQWSVPELPSPPTASGLHWQRRPAAHGASDSTAFVPDVCSPDNSLRYCFPADQPVTAKCRRKC |
| PH02Gene23282.t1 | PeBBX13 | MKIGCDACGQAEAAVLCCADEAALCRRCDAAVHSANKLAGKHHRVALLPSGGGLSPACSSSPDADDGGHPVCDICQEKRGYFFCLEDRALLCRSCDVAVHTTSPYVSTHRRFLITGVRIGGAQDHIPGSDVSGTAGAVVSSSSSSGDGSNNLPSSSNLIIPDNGRPSEAGLGEEEDIGRQQQWPWSDIFADGVGMEQCYPGLSEPGSSSLTG |
| PH02Gene32457.t1 | PeBBX14 | MKVLCSACEAAEARVLCCADEAALCARCDRDVHAANRLAGKHHRLPLLSPTSAATTVSAPKCDICQECHAYFFCVEDRALLCRSCDVAVHTANAFVSAHRRFLLTGVQVGLVPDDQEPEPPNASAAPPMQPPPCPKKSSPTLLYSDDDIDWAAGPDVGITRNLPDWSVVDEQFSSPALRPAEPVISKTPPKRSPRGPVTAGSAAVFGGLGGVAGSMHDWPLDEFFGFAEFNAGFGFTENGTPNADSAKIGSAYGSPNRRSSSSDGAATQNAQDFFGQVPEFHWSVPELPSPPTASGLHWQGDPHYGAAATDTAVFVPDICSPENPFRCFPTAGQPQSAHLKRRRRC |
| PH02Gene09921.t1 | PeBBX15 | MTKAGAATGAVLGARTARACDSCMRRRARWHCAADDAFLCQACDASVHSANPLARRHHRVRLPSASSSPASFPPRADDPDAPAWLHGLKRRPRTPRPKPGSGKHEASAIATMVAAASAVPDLGAEESGIVGDNDVEDDDEDLLYRVPVFDPMLAELYNPVPDEGEPLEQKPTCLASLAEPTPAFASGSAEANGLSGFFVPDMELASFAADMESLLMGVDDGFDNLGFLDDEKPQVNVDLDTMAAPGPEREDRKKKRPGMMLKLDYDRVISSWTRDGGSPWFHGERPHLDQGESWPDFPAGSRGGLSAVVTAVTGGEREARVSRYREKRRTRLFAKKIRYEVRKLNAEKRPRMKGRFVKRTALPLSRPPPRALPHHAQVPMVLAPHIAHGRFRF |
| PH02Gene20422.t1 | PeBBX16 | MKVLCSACEAAEARVLCCADEAALCARCDRDVHAANRLAGKHHRLPLLSPASAAAAVSAPKCDICQECHAYFFCVEDRALLCRSCDVAVHAANAFVSAHRRFLLTGVQVGLEPDDQEPEPPQPPNASAAAPLQPPPCPKKSSPTPLYSDDDIDWAAGPDVGITGNLPDWSVVDEQFSSPALRPAEPVVSKTPPKRSPRGPITAGSAAVFDGGMPDWPLDEFFGFAEFNAGFGFAENGTSKADSAKLGSTDGSPNHRSSFSDCAATQNAQDFFGQVPEVHWSVPELPSPPTASGLHWQGNPHYGASGATDTALFVPDICSPENPFRCFPTASQPQPAHLKRRRRS |
| PH02Gene08112.t1 | PeBBX17 | MSSSEKAAGAVGGKAARACDSCLRRRARWYCAADDAFLCQGCDTSVHSANPLARRHERLRLRASFPPSPPPQSVEGVAAATTSKRQGISLAWSKRKARTRRPQVKSVGQLLSRRPRLIVPEVAVESSEERKPEEDGAEEEQFLFRVPIFDPALAEFCSPPPIDDAAATASCCREDVDGALENTREAVVAASPVQQLPDSLANFGPTDAELRVFAADMEALLGRGLDDSNELDDPFYVENLGLVSPAEDGGRVKVEPGGVVSNSGGAQASGPELKSEASAEVLDIHFNCSSPTVVDDNDGFEQKTSASKGDAADAQFFQRSLDLRLNYEAVIESWGSSPWTDGRRPHVQLDDFRLHDHPGMWTAGGGRHGEEAGTPKPRMDGAGREARVTRYREKRRTRLFAKKIRYEVRKLNAEKRPRMKGRFVKRPAGGGAAVAVAVAAAPCAVT |
| PH02Gene14035.t1 | PeBBX20 | MKIQCDACEGAAATVVCCADEAALCARCDVEIHAANKLASKHQRLPLDALGARLPRCDVCQEKAAFIFCVEDRALFCRDCDEPIHVPGTLSGNHQRYLATGIRVGCGSVSTCSGATNSAHDADHHAPPKATSEHPPPALAAAAVAQQVPSPPQFLPQGWAVDELLQFSDYESSDKLQKESPLGFKELEWFADIDLFHDQAPKGGRTMVEVPELFASHAVNDAAYYRPSRGAGVRQSKKPRVDIPDDEDDYLIVPDLG |
| PH02Gene28332.t1 | PeBBX19 | MDAEDRKPVAGAGCWGLGARPCDTCGVDAARLYCRADGAFLCPGCDARTHGAGSRHARVWLCEVCEHAPAAVTCRADAAALCAACDADIHSANPLARRHERLPVAPFLGALADAPQPFPSSAFAAAVAATEAQEDADDDGSNEAEAASWLLPEPDKGHEDSAAADAFFADSDAYLGLDLDFARSMDGIKAIGVLAAQPELDLAAGNLFYPEHSMNHSVSSSEVAVVPDALSAGAAPAPSLAVVASKGKEREARLMRYREKRKNRRFDKTIRYASRKAYAETRPRIKGRFTKRTTEDDALLEHDRACSPAMSALVASDGDYGVVPSF |
| PH02Gene29322.t1 | PeBBX18 | MKVQCDVCAAEAASVFCCADEAALCDACDRRVHRANKLAGKHRRFSLLNPPPYSSASENPPPPPLCDICQERRGFLFCKEDRAILCRECDVPVHTASELTMRHNRFLLTGVRLSSEPAASPAPPSEEENSSSFCCSGDDAAPPAPATSHGGSSGSSISEYLTKTLPGWHVEDFLVDDASAAAAAAATSTGISADGSYQGVTRIGGLQEAGYHAWMAQEQLFCDSVVAGDALASRERWVPQMMYTELAGSKRSRTSASNSYW |
| PH02Gene08231.t1 | PeBBX21 | MKVLCSACEAAEARVLCCADEAALCARCDRDVHAANRLAGKHQRLPLLTPAATPVSLPPKCDICQECYAYFFCLEDRALLCRSCDVAVHTANAFVSAHRRFLLTGVQVGQEEDDHSPDPPEPSPPPPAKSDPAPPYGEGDFSWAAATPDATGVTGGLPDWSIVNEQFGSPAPRHTEVANRTRPKRSPRAPAFGGQGGMMDWPLGEFFGGFSDFNGSFGFGESGTSKADSGKLGGSTDGSPYYRSSSEDRNAVELFGQVPEIQWSVPELPSPPTASGLHWQRRPAAHGASDSTAFVPEICSPDNSLRYCFPAGQPATAKRRRK |
| PH02Gene13905.t1 | PeBBX22 | MTDAGAATGAALGARTARACDGCMRRRARWHCEADDAFLCQACDASVHSANPLARRHHRVRLPSASTSPASSPPRSADPDAPAWLHGLKRRPRTPRSKPGGGKHEASTAASAVPDLEADESGVVGDNDVEDDDEYLLYRVPVFDPMLAELYNPVPDEGEPLEQKPACLASLAEPSPEFASGSAEADGLSGFDVPDMELASFAADMESLLMGVDDGFDDLGFLDEEKPQVNLELDMDFDTMAAPLPELVDSKKKRPEMILKLDYEGVISSWARDGGSPWFHGERPHLDHGESWPEFPAGSRGVLGAAVTSGEREARLSRYREKRRTRLFAKKIRYEVRKLNAEKRPRMKGRFVKRTALPPLPLSRPPPPPPRALPHHAQVPMVLAPHVAHGRFRF |
| PH02Gene06669.t1 | PeBBX24 | MEDDEKSVGGAYWGLAAKACDACGAEAARLFCRADAAFLCAGCDARAHGPGSRHARVWLCEVCEHAPAAVTCRADAAALCVTCDADIHSANPLARRHERLPVAPFFGALADAPKPFASSGLGGGAGAHGTAAADDDGSNEAEAASWLLPEPDHGQQKDGPVGATDVLFADSDPYLDLDFARSMDDIKAIGVQNSLPELDLTGAKLFCSDHSMNHSVSSSEAAVVPDAAAGAAPVPVVSKGREREARLMRYREKRKSRRFEKTIRYASRKAYAETRPRIKGRFAKRTTGAQAEADADGEDAMEHEEMYSSAAAAVAALMAPGPDADYGVVPTF |
| PH02Gene11378.t1 | PeBBX25 | MRIQCDACEGAAATVVCCADEAALCARCDVEIHAANKLASKHQRLPLDAALPAALPRCDVCQEKVAFIFCVEDRALFCRDCDEPIHVPGTRSGNHQRYLATGIRVGFGSVCSANVGTHADHLPSKGSSKPPSVVAAGGVPKRVPAAAQEVPSSPFLPPSGWAVEDLLQLSDYESSDKKESPLGFKELDWFEDIDLFHDHAPGKWGSTAPEVPELFASPQPASNAGFYKTSGERQSKKSRVELPDDDEDYLIVPDLG |
| PH02Gene35510.t1 | PeBBX23 | MKVQCDVCAGEAASVFCCADEAALCDACDRRVHRANKLAGQHRRFSLLHPSSSSTQKPPLCDICQEKRGFLFCKEDRAILCRECDVSVHTASELTRRHSRFLLTGVRVSSAPVGSPALSSGEEAEEENSSSPCNADSCSGAGTATASARDGSSISEYLTKTLPGWHVEDFLMDDAAAAVSPDEPYQGGGLDQIGGLQEGYTAWAGREQLLSDVVVAADERASREWWVPQMHAEWAGSKRPRASPPHSYW |
| PH02Gene02031.t1 | PeBBX27 | MKVQCDVCAAEAASVFCCADEAALCDACDRRVHRANKLAGKHRRFSLLYPSSSFAQKPPLCDICQEKRGFLFCKEDRAILCRECDVPVHTASELTRRHSRFLLTGVRVSTALVDSPAPSIEEAEEENSSSPCNADSCSGAGAATPNASDGSSISEYLTKTLPGWHVEDFLMDDAAASAAVTVSPDEPYQVGGLSQIGGLQEGYPAWEDREQLLSDVVVAADERTSWERWVPQMHAEWAGSKRPRASPPHPYW |
| PH02Gene03202.t1 | PeBBX26 | MRIQCDACEGAAATVVCCADEAALCARCDVEIHAANKLASKHQRLPLDAALPAALPRCDVCQEKSAFIFCVEDRALFCRDCDEPIHVPGTLSGNHQRYLATGIRVGFGSVCNANVGTRADHLPPKGGSKPPSVVAAGGVPNPVPAAAQEVPSLPFLPPSGWAVEDLLQFSDYESSHKESPLGFKELEWFADIDLFHDHAPAMGGSTAAEVPELFASPQPASNAGFYKTSGARQSKKRRVELPDDDEDYLLVPDLG |
