# Supplementary material for: The BBX gene family in Moso bamboo (Phyllostachys edulis): identification, characterization and expression profiles
Source: BMC Genomics. 2021 Jul 13;22:533. doi: 10.1186/s12864-021-07821-w (PMC8276415; doi:10.1186/s12864-021-07821-w)
Supplement: Supplementary file 7 — Additional file 7: Table S4. The primers used for qRT-PCR. [file 12864_2021_7821_MOESM7_ESM.docx]

**S4 Table. The primers used for qRT-PCR**

| **Primer Name** | **Sequence** |
| --- | --- |
| ACTF | ATCCAGAAGGAGCGAGTTTG |
| ACTR | ACTGGGGTCCGTCTGTAATG |
| PeBBX07F | GGATGGTGGGAGGAGGAGTGGTTG |
| PeBBX07R | GCGTACCTCGTACCGGATCTTCTTGG |
| PeBBX14F | TTCACCGAGAATGGCACGCCAAAT |
| PeBBX14R | CCGGCACGGACCAGTGGAACT |
| PeBBX15F | AAAACGTCCGGGGATGATGCTTAAG |
| PeBBX15R | CTCGCCTTGGTCGAGGTGAGGG |
| PeBBX17F | CTGGCATGGTCGAAGCGGAAGG |
| PeBBX17R | GGGTCAAAGATCGGTACGCGAAACA |
| PeBBX20F | TAACAAGCTCGCCAGCAAGCACCA |
| PeBBX20R | GCCCTGTCCTCCACGCAGAAGATG |
| PeBBX25F | GGGTTCAAGGAGCTGGACTGGTTCG |
| PeBBX25R | TGACTTCTTGCTCTGGCGCTCACC |
| PeBBX26F | ACAAGCTCGCCAGCAAGCACCA |
| PeBBX26R | GCCCTGTCCTCCACGCAGAATATGAA |
